# Combating VEGFA‐siRNA‐Induced Metabolic Reprogramming via Glucose Utilization Deprivation

**DOI:** 10.1002/advs.202519290

**Published:** 2026-04-09

**Authors:** Lulu Zheng, Shuai Guo, Yingjixing Luo, Pengfei Wu, Haiyin Yang, Yingqiu Xie, Yuchuan Fan, Qing Liu, Bo Hu, Jia Huang, Yuanyu Huang

**Affiliations:** ^1^ School of Life Science School of Interdisciplinary Science Key Laboratory of Molecular Medicine and Biotherapy Key Laboratory of Medical Molecule Science and Pharmaceutics Engineering Beijing Institute of Technology Beijing China; ^2^ Department of Hepatobiliary Surgery China‐Japan Friendship Hospital Beijing China; ^3^ Department of Biology School of Sciences and Humanities Nazarbayev University Astana Kazakhstan; ^4^ State Key Laboratory of Chemical Resource Engineering College of Chemical Engineering Beijing University of Chemical Technology Beijing China; ^5^ School of Medical Engineering School of Interdisciplinary Science Affiliated Zhuhai People's Hospital Beijing Institute of Technology Zhuhai China; ^6^ Advanced Technology Research Institute Beijing Institute of Technology Jinan China; ^7^ Zhengzhou Research Institute Beijing Institute of Technology Zhengzhou China

**Keywords:** glucose oxidase, glutamine metabolism, lipid nanoparticle, tumor metabolic reprogramming, VEGFA siRNA

## Abstract

Vascular endothelial growth factor (VEGF) inhibitors suppress tumor energy supply, but their efficacy is often limited by the restoration of tricarboxylic acid (TCA) cycle activity and enhanced glycolysis. Here, a synergistic strategy is established using an in‐house‐designed ionizable lipid nanoparticle (LNP) to co‐encapsulate VEGFA‐targeting siRNA (siVEGFA) and glucose oxidase (GOx), thereby enhancing siRNA efficacy by depleting both aerobic and anaerobic glucose utilization. At the cellular level, the optimal formulation, iVG128, inhibits energy production, suppresses microvessel formation, and induces mitochondrial ultrastructural changes, leading to persistent suppression of the TCA cycle. In both CT26 cell‐derived and patient‐derived xenograft tumor models, iVG128 shows potent antitumor activity, achieving 2.6‐fold higher efficacy than Sorafenib, significantly prolonging survival. Untargeted metabolomics indicates that iVG128 eliminates the glutamine‐driven compensation induced by VEGF inhibition, thereby exacerbating metabolic stress and promoting apoptosis. Transcriptomic profiling reveals that VEGFA silencing induces adaptive gene programs related to PDH inhibition, hypoxia signaling, and glutamine metabolism, and these responses are largely suppressed by iVG128. Collectively, iVG128 represents a versatile nanoplatform for co‐delivering enzymatic and RNA therapeutics, offering an effective strategy for cancer treatment through energy source depletion.

## Graphical abstract

An ionizable lipid nanoparticle co‐delivers VEGFA siRNA and glucose oxidase to tumors to counteract VEGFA knockdown‐induced, glutamine‐driven metabolic compensation. Enzymatic glucose consumption reverses the adaptive pathways and restores metabolism toward baseline, thereby sensitizing tumors to therapy. The synchronized dual cargo yields durable tumor growth suppression with low systemic toxicity.

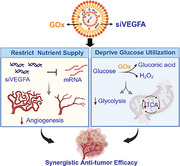

## 1 Introduction

Tumor cells require substantial energy and nutrients to sustain rapid proliferation [1], rendering anti‐angiogenic therapy a promising approach for cancer treatment [2]. Vascular endothelial growth factor (VEGF) is a well‐known driver of tumor angiogenesis [3], which promotes endothelial cell proliferation and migration through receptor‐mediated activation of downstream signaling pathways [4]. Among VEGF family members, VEGFA is the predominant isoform, initially identified as a regulator of endothelial growth and vascular permeability [5]. To date, multiple VEGF inhibitors have been clinically applied for the treatment of various malignancies, including hepatocellular carcinoma (HCC) and colorectal cancer (CRC) [6]. For instance, bevacizumab, the first FDA‐approved anti‐angiogenic agent, has demonstrated remarkable efficacy across a broad range of cancers and has been approved for multiple indications [7]. Similarly, Sorafenib has demonstrated remarkable efficacy in the treatment of advanced hepatocellular carcinoma by effectively targeting VEGFR to inhibit tumor angiogenesis [8, 9]. Despite these successes, long‐term VEGF inhibition frequently triggers metabolic reprogramming that limits therapeutic efficacy. Patients with long‐term exposure to bevacizumab have been found to develop a dependence on glutamine metabolism [10]. Additionally, a study has reported that angiogenesis inhibitors led to an upsurge in glycolysis within tumor tissues [11]. These findings underscore that adaptive upregulation of glycolysis or the tricarboxylic acid (TCA) cycle may ultimately lead to therapeutic failure [12, 13].

Glycolysis and the TCA cycle are central pathways in cellular energy metabolism [14, 15]. In these processes, glucose undergoes sequential oxidation through glycolysis and the TCA cycle to generate adenosine triphosphate (ATP), which is essential for sustaining cellular viability [16, 17]. In tumors, aberrant reliance on glycolysis facilitates uncontrolled proliferation and invasion [18]. However, given the intrinsically low ATP yield of glycolysis, tumor cells are particularly vulnerable to glucose deprivation [19]. Glucose oxidase (GOx) converts β‐D‐glucose into gluconic acid with the concurrent production of hydrogen peroxide (H_2_O_2_), thereby disrupting both glycolysis and the TCA cycle [20, 21, 22]. Although GOx‐mediated “starvation therapy” has attracted significant attention [23], its potential role in reversing resistance to VEGF inhibition has been largely unexplored.

**SCHEME 1:**
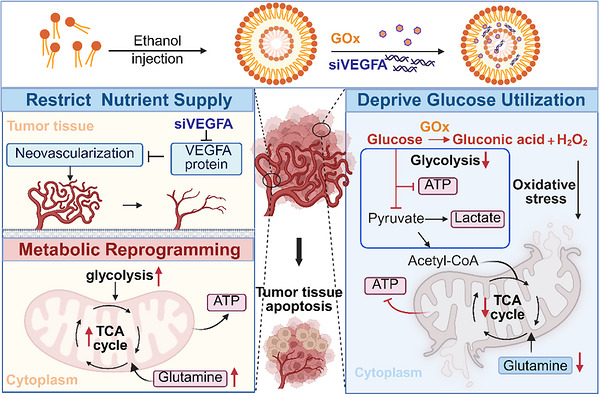
The strategy illustration of this work. siVEGFA inhibits angiogenesis and restricts nutrient supply, but induces compensatory metabolic reprogramming. The introduced GOx not only suppresses glycolysis by consuming intracellular glucose but also generates H_2_O_2_ to block the TCA cycle by damaging mitochondrial ultrastructure. This dual metabolic intervention synergistically disrupts tumor metabolic plasticity, ultimately leading to metabolic collapse and tumor suppression.

Here, we propose a strategy for reversing anti‐angiogenic therapy‐induced metabolic reprogramming by depriving tumors of glucose utilization (Scheme 1). Chemically modified small interfering RNA (siRNA) targeting VEGFA (siVEGFA) was employed as a cost‐effective alternative to expensive VEGF antibodies. It was co‐encapsulated with GOx in an ionizable lipid‐based nanoparticle (iLAND), resulting in a lead formulation iVG128. Both active ingredients with distinct properties were efficiently co‐loaded into iLAND, and the resulting formulation retained the excellent stability and high delivery efficiency of iLAND. In both CT26 cell‐derived xenograft (CDX) and patient‐derived xenograft (PDX) tumor‐bearing models, monotherapy with GOx‐loaded nanoparticles (iNG128) or siVEGFA‐loaded nanoparticles (iV) elicited measurable tumor inhibition. Notably, iVG128 demonstrated robust and consistent tumor‐suppressive activity, which outperformed the clinically‐used anticancer small molecule Sorafenib.

Untargeted metabolomics revealed that iV treatment activated compensatory TCA cycle pathways, as indicated by elevated glutamine and L‐malic acid levels, which is consistent with clinical resistance mechanisms. To further understand the impact of GOx in iVG128 on tumoral metabolic reprogramming, transcriptomics profiling was carried out. The results indicated that it effectively normalized the levels of PDH gate regulators (*Pdk2*/*Pdk4*), HIF/hypoxia signaling, glutamine transport/utilization (*Slc38a3*/*Gls*), and carbon recycling/gluconeogenesis‐like metabolic rewiring (*Pck1*/*G6pc*/*Aldob*), which were upregulated by iV. Collectively, this study establishes iVG128 as a multifunctional nanoplatform capable of co‐delivering enzymatic and RNA therapeutics, and underscores the underappreciated potential of GOx to mitigate metabolic compensation during VEGF‐targeted therapy.

## 2 Results

### 2.1 Formulation Optimization for Co‐Delivery of siRNA and GOx

In our previous work [24], we determined that an iLAND‐to‐siRNA mass ratio of 15:1 provided optimal delivery performance. This ratio was therefore adopted in the current work. To further optimize co‐delivery, we systematically screened formulations by varying the iLAND‐to‐GOx mass ratio from 8:1 to 256:1, while maintaining a constant siRNA‐to‐GOx volume ratio of 1:1. Here, the siRNA against firefly luciferase was used as negative control (recorded as siNC). For clarity, these formulations were designated as iNG8 to iNG256.

**FIGURE 1:**
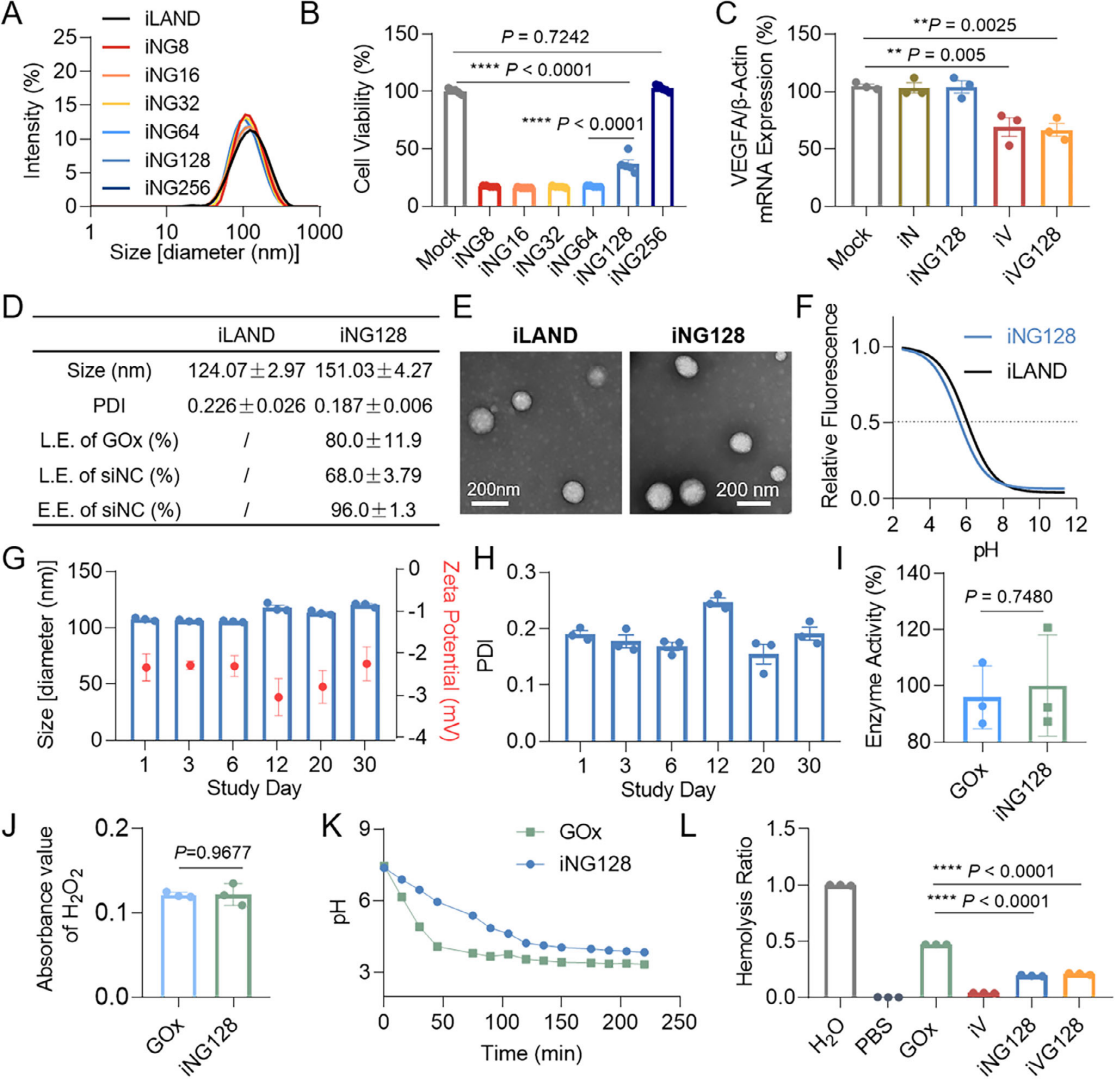
Screening and physicochemical characterization of iNG128. (A) Hydrodynamic diameters of iNG8 to iNG256, measured by dynamic light scattering (DLS). (B) Cell viability of CT26 cells treated with different formulations, evaluated by CCK8 assay, siRNA concentration at 100 nm. (C) Quantitative PCR analysis of VEGFA expression in CT26 cells, siRNA concentration at 100 nm. (D) Physicochemical properties of iLAND, such as particle size, PDI, zeta potential, encapsulation efficiency (E.E.), and loading efficiency (L.E.), were evaluated before and after loading with siNC and GOx. (E) Representative TEM images of iLAND and iNG128. (F) The pKa values of iLAND and iNG128. (G) The long‐term stability of iNG128 at 4 °C was evaluated by measuring particle size and zeta potential over 30 d. (H) Measurement of PDI of iNG128 during storage. (I) Comparison of enzymatic activity between free GOx and iNG128. (J) H_2_O_2_ production in glucose solution, catalyzed by free GOx or iNG128. (K) pH change of glucose solutions after treatment with iNG128 or free GOx. (L) Hemolysis assay of free GOx or LNP formulations, PBS and H_2_O were included as negative and positive controls, respectively. *n* = 3 biologically independent experiments. All quantitative results are presented as the mean ± standard error of mean (S.E.M.). *p*‐value < 0.05 was considered statistically significant, with *p < 0.05, **p < 0.01, ***p < 0.001, and ****p < 0.0001.

Dynamic light scattering (DLS) analysis revealed that GOx incorporation had minimal influence on particle size (Figure 1A), indicating that GOx is compatible with the iLAND delivery platform. We then assessed the in vitro antitumor activity of these formulations. The results showed that iNG8, iNG16, iNG32, and iNG64 induced approximately 83% cell death, iNG128 induced 62.8% cell death, while iNG256 exhibited minimal cytotoxicity (Figure 1B). Next, siNC was replaced with siVEGFA to evaluate the gene silencing efficiency of formulations. Given the lack of antitumor activity observed in iNG256, only formulations from iVG8 to iVG128 were prepared for this analysis. The excessive cytotoxicity of iNG8 to iNG64 resulted in insufficient cells for assessing gene suppression efficiency, whereas iVG128 mediated 33.3% VEGFA gene silencing, which was comparable to the performance of iV (without GOx encapsulation) (Figure 1C). Based on these findings, iVG128 was selected as lead formulation, owing to its balanced antitumor efficacy and efficient gene silencing capability.

### 2.2 Basic Physicochemical Characterization of iNG128

We next systematically characterized the physicochemical properties of iNG128. DLS revealed that iLAND exhibited an average particle size of 124.07 nm with a polydispersity index (PDI) of 0.226, whereas co‐encapsulation of GOx and siRNA increased the size to 151.03 nm and slightly decreased the PDI to 0.187 (Figure 1D). GOx and siRNA loading efficiencies (L.E.) were 80.0% and 68.0%, respectively. The siRNA encapsulation efficiency (E.E.) reached 96.0%, confirming the suitability of iLAND for co‐delivery. TEM images further revealed well‐defined lipid bilayers and uniform particle morphology for both iNG128 and iLAND (Figure 1E).

It has been reported that the pKa of ionizable lipids is a critical factor for effective endosomal escape, with an optimal value above 5.5 [25]. In our previous study, the pKa of iLAND was determined as 6.16, meeting this criterion. Here, we assessed whether GOx incorporation influences this parameter using 2‐(p‐toluidino)‐6‐naphthalene sulfonic acid (TNS) as a probe. We found that the pKa decreased slightly from 6.07 to 5.66 upon GOx encapsulation (Figure 1F), but remained sufficient to support endosomal/lysosomal escape. Storage stability tests showed that iNG128 maintained consistent particle size, zeta potential (Figure 1G), and PDI (Figure 1H) over 30 days at 4°C, confirming excellent formulation stability.

### 2.3 Enzymatic Activity of the Encapsulated GOx

To assess whether encapsulation affected GOx activity, we compared iNG128 with free GOx at equivalent concentrations in glucose‐containing solutions. Both enzymatic activity (Figure 1I) and H_2_O_2_ production (Figure 1J) were comparable, indicating that GOx activity was fully preserved. Continuous pH monitoring showed similar final acidification levels between groups, confirming equivalent gluconic acid production. Interestingly, it was observed that iLAND delayed the pH drop (Figure 1K). We believe that a smaller pH change within the same period contributes to a better safety profile. To prove this, we performed a hemolysis test to evaluate the safety of iV, iNG128, iVG128 and free GOx. After treating the corresponding formulations with blood from healthy mice, the absorbance was measured to reflect hemolysis (Figure 1L). It was found that free GOx significantly triggered hemolysis, whereas iV did not, demonstrating the excellent safety of iLAND. Importantly, we noticed that though the encapsulated GOx still triggered hemolysis, the measured absorbance was significantly lower than that of GOx, proving that encapsulation in iLAND enhanced the safety of GOx. Collectively, these findings demonstrate that iLAND fully preserves the activity of GOx and substantially enhances the biocompatibility of GOx by controlling its release.

### 2.4 Internalization Behavior of iNG128

**FIGURE 2:**
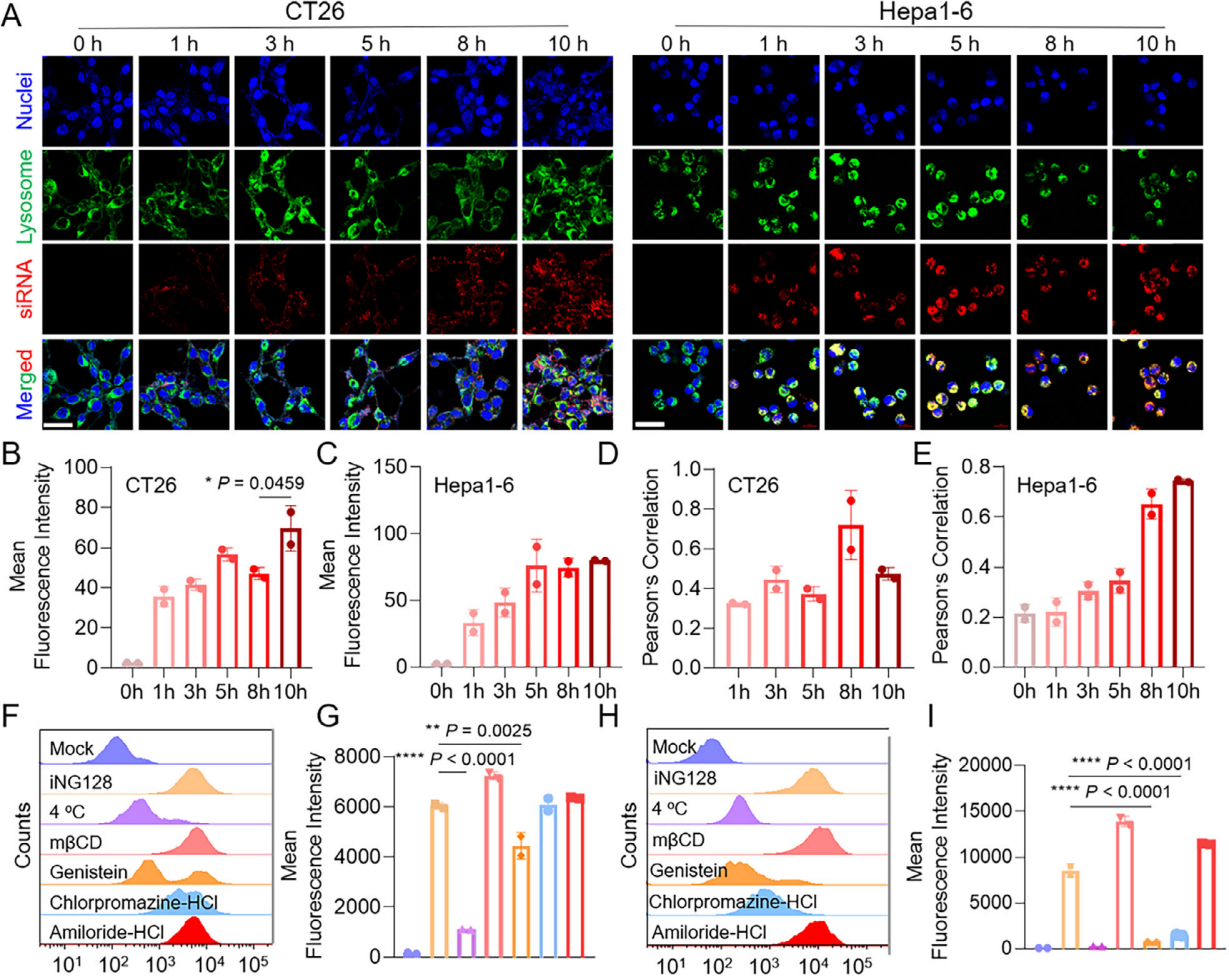
Subcellular localization and cellular uptake mechanism of iNG128. (A) Subcellular localization of iNG128 in CT26 and Hepa1‐6 cells at different transfection time points revealed by CLSM images. Scale bars: 40 µm. (B) Quantification of average fluorescence intensity from CLSM images in CT26 cells, and (C) Hepa1‐6 cells. (D) Colocalization between Cy5‐siRNA and endosomes/lysosomes in CT26 cells, and (E) Hepa1‐6 cells. (F) Quantification of intracellular Cy5‐siRNA signal in CT26 cells pretreated with different endocytosis inhibitors by FACS analysis. (G) Quantification of average fluorescence intensity of Cy5 in CT26 cells. (H) Quantification of intracellular Cy5‐siRNA signal in Hepa1‐6 cells pretreated with different endocytosis inhibitors by FACS analysis. (I) Quantification of average fluorescence intensity of Cy5 in Hepa1‐6 cells. All quantitative results are presented as the mean ± S.E.M. (*n* = 2). *p*‐value < 0.05 was considered statistically significant, with *p < 0.05, **p < 0.01, ***p < 0.001, and ****p < 0.0001.

Although the pKa of iNG128 suggests efficient endosomal/lysosomal escape, direct confirmation of cytoplasmic delivery is essential. To this end, Cy5‐labeled siNC (Cy5‐siNC) and GOx were co‐encapsulated into iLAND to generate Cy5‐iNG128. CT26 and Hepa1‐6 cells were used to track intracellular trafficking over 10 h via confocal laser scanning microscopy (CLSM). Cy5 fluorescence progressively accumulated in both cell types, indicating efficient cellular uptake (Figure 2A). Multi‐field quantitative analysis corroborated these observations (Figure 2B,C). To characterize the intracellular trafficking of iNG128, we analyzed the colocalization between Cy5‐labeled siRNA and endosomes/lysosomes using Pearson's coefficient. In CT26 cells (Figure 2D), the Pearson's coefficient increased after transfection and reached a maximum at approximately 8 h, indicating that a substantial fraction of internalized siRNA was localized within endosomal/lysosomal compartments during this period. Notably, at later time points, the Pearson's coefficient gradually decreased. This temporal pattern suggests a dynamic redistribution of siRNA, in which the rate of siRNA release from endosomal/lysosomal compartments exceeds the rate of further endocytic accumulation after prolonged incubation, leading to a relative reduction in colocalization.

In contrast, Hepa1‐6 cells (Figure 2E) showed a different colocalization profile, and the temporal trend was not identical to that observed in CT26 cells. This discrepancy indicates that the intracellular trafficking and redistribution of siRNA are cell‐type dependent, consistent with differences in endocytic processing and intracellular transport pathways among distinct cell lines.

### 2.5 Cellular Uptake Mechanism of iNG128

To dissect the pathways mediating iNG128 uptake, CT26 cells were incubated with specific uptake inhibitors prior to treatment: chlorpromazine‐HCl (clathrin‐mediated endocytosis), amiloride‐HCl (macropinocytosis), genistein (caveolae‐mediated endocytosis), and methyl‐β‐cyclodextrin (mβCD, migrasome‐mediated endocytosis) [26]. Cells were then incubated with Cy5‐iNG128 for 4 h at either 37°C or 4°C, followed by intracellular fluorescence quantification via flow cytometry. As shown in Figure 2F,G, incubation at 4°C or genistein pretreatment significantly inhibited the endocytosis of iNG128 by CT26 cells, indicating that the internalization is energy‐dependent and primarily involves the caveolae‐mediated endocytic pathway. Interestingly, both clathrin‐ and caveolae‐mediated pathways are involved in the internalization of iNG128 in Hepa1‐6 cells (Figure 2H,I). These findings suggest that the endocytic mechanisms of iNG128 uptake are cell‐type dependent.

### 2.6 GOx Catalysis‐Driven Metabolic Disruption and Mitochondrial Dysfunction

**FIGURE 3:**
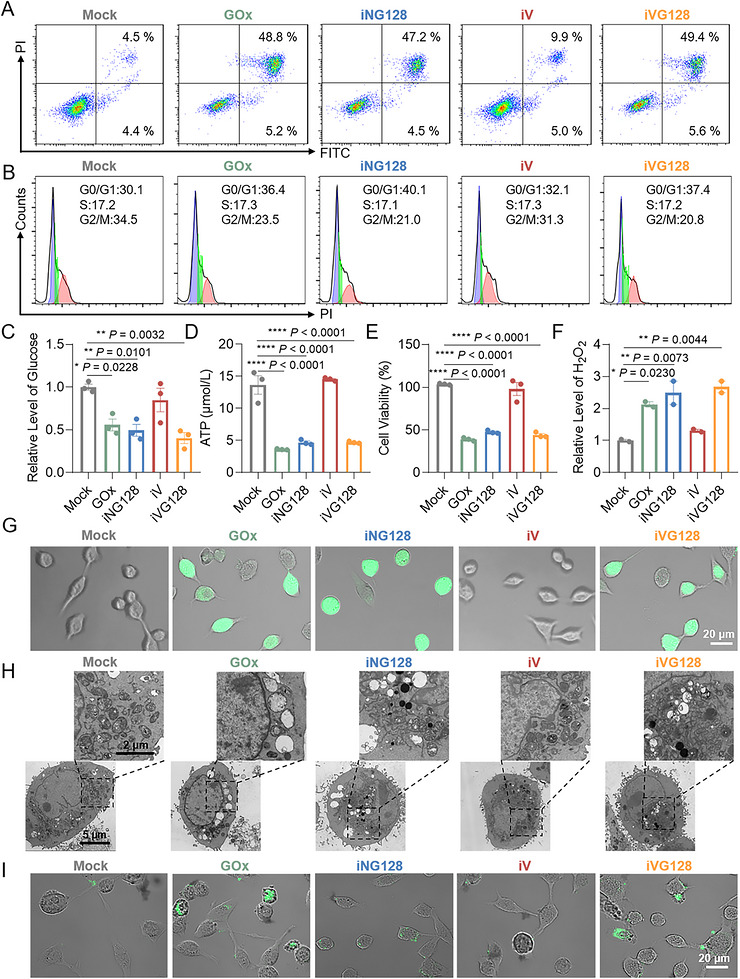
In vitro evaluation of antitumor efficacy of iVG128. (A) Early and late apoptosis in CT26 cells detected by FACS after 12 h transfection. (B) Cell cycle of CT26 cells detected by FACS after 12 h transfection. (C,D) Intracellular glucose and ATP levels in CT26 cells. (E) Cell viability assessed by CCK8 after 12 h transfection. (F) Intracellular H_2_O_2_ levels in CT26 cells after different treatments. (G) Intracellular ROS in CT26 cells visualized by confocal microscopy. (H) Representative bio‐TEM images of mitochondria in CT26 cells after different treatments. (I) Mitochondrial membrane potential (ΔΨm) in CT26 cells assessed by JC‐1 staining. Quantitative data are presented as mean ± S.E.M., with *n* = 3 biologically independent samples for (A–E, G–I) and *n* = 2 biologically independent samples for (F). *p*‐value < 0.05 was considered statistically significant, with *p < 0.05, **p < 0.01, ***p < 0.001, and ****p < 0.0001.

Next, in vitro assays were performed to evaluate the cytotoxic effects of iVG128, starting with apoptosis analysis. In untreated cells, only a small fraction of cells underwent apoptosis (early apoptosis 4.4%, late apoptosis 4.5%), whereas free GOx induced late apoptosis in nearly half of the cells (48.8%). iV induced negligible apoptosis, but both iNG128 and iVG128 induced levels of apoptosis comparable to free GOx, with 47.2% and 49.4% late apoptotic cells, respectively (Figure 3A). Cell cycle analysis revealed that GOx, iNG128, and iVG128 all induced pronounced G0/G1 arrest, consistent with their antiproliferative effects, whereas siVEGFA alone had no detectable impact (Figure 3B and Figure S1).

To more convincingly demonstrate that the downstream effects observed in Figure 3A are indeed attributable to the catalytic activity of GOx, we directly measured changes in intracellular glucose levels in tumor cells after treatment. As expected, the groups containing active GOx (free GOx, iNG128, and iVG128) significantly reduced intracellular glucose content, showing marked depletion compared with the untreated and iV groups (Figure 3C). Afterwards, we tried to explore whether the cytotoxic effects of GOx were attributed to the alteration in cellular metabolism. Intracellular lactate and ATP were quantified as indicators of glycolytic flux and energy status. iVG128 significantly reduced both lactate (Figure S2) and ATP (Figure 3D) levels, consistent with potent inhibition of glycolysis and energy production. Free GOx and iNG128 induced similar metabolic impairments, whereas iV had negligible effects. Importantly, the extent of metabolic suppression correlated with cell death across treatment groups (Figure 3E).

To confirm metabolic impairment, the level of H_2_O_2_ was determined. Figure 3F showed that GOx, iNG128, and iVG128 treatments markedly elevated H_2_O_2_ levels, indicating a high oxidative stress environment. Consistent with these findings, confocal imaging using a ROS‐sensitive fluorescent probe showed a marked increase in intracellular ROS fluorescence signals in the GOx‐containing treatment groups (Figure 3G and Figure S3). Biological TEM images showed intact mitochondrial cristae in untreated and iV‐treated cells, whereas GOx‐, iNG128‐, and iVG128‐treated cells displayed cristae loss and vacuolization (Figure 3H), indicating severe mitochondrial injury. To further verify whether this structural damage is accompanied by mitochondrial functional failure, we assessed the mitochondrial membrane potential (ΔΨm) using JC‐1 staining. Compared with the control and iV groups, GOx, iNG128, and iVG128 all caused a pronounced loss of ΔΨm, as indicated by increased JC‐1 green fluorescence (Figure 3I and Figure S4). Collectively, the ultrastructural disruption of mitochondria together with the collapse of membrane potential provide direct evidence of mitochondrial dysfunction, which is expected to further compromise oxidative phosphorylation (OXPHOS) and TCA cycle activity, thereby precipitating an energy crisis and promoting cell death.

### 2.7 iVG128 Exhibits Potent Anti‐Angiogenic Activity

**FIGURE 4:**
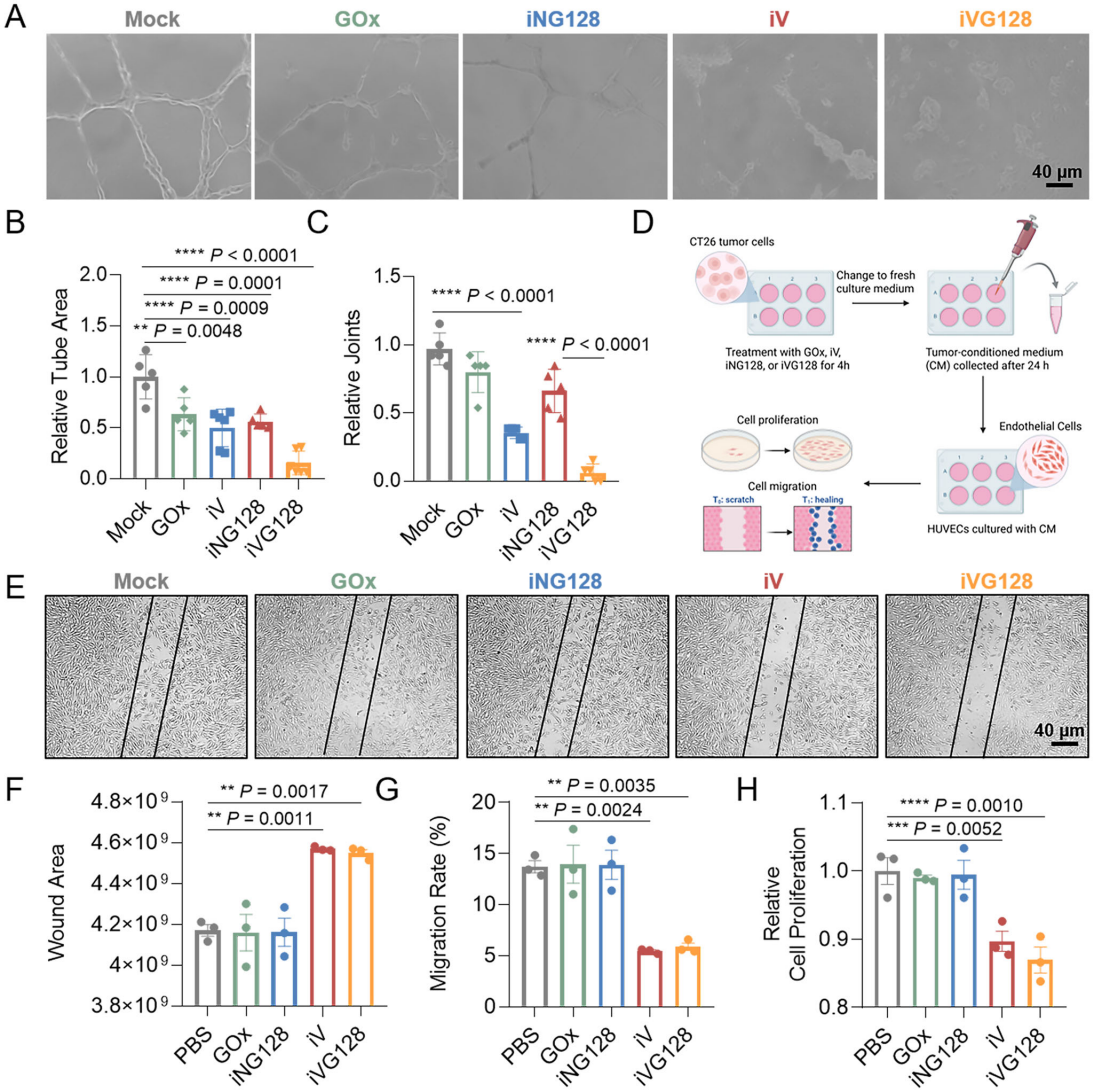
In vitro evaluation of anti‐angiogenic activity of iVG128. (A) Tube formation of HUVECs after different treatments. (B) Quantitative analysis of HUVEC tube area. (C) Quantitative analysis of HUVEC relative joints. (D) Schematic illustration of the workflow for HUVEC proliferation and migration assays, in which the medium was replaced with fresh medium after 4‐hour transfection to minimize the direct cytotoxicity of GOx, and HUVECs were subsequently cultured with tumor cell supernatants collected after 24‐hour treatments. (E) Scratch wound migration assay of HUVECs after different treatments (representative images at 48 h). (F) Quantitative analysis of wound area in the scratch assay. (G) Migration rate of HUVECs calculated based on the wound area at 0 h. (H) HUVEC proliferation assessed by CCK‐8 after incubation with conditioned media. Quantitative data are presented as mean ± S.E.M., with *n* = 5 biologically independent samples for (A–C) and *n* = 3 biologically independent samples for (E–H). *p*‐value < 0.05 was considered statistically significant, with *p < 0.05, **p < 0.01, ***p < 0.001, and ****p < 0.0001.

In addition to directly inducing tumor cell death, we further evaluated the anti‐angiogenic activity of iVG128. In an in vitro tube formation assay, human umbilical vein endothelial cells (HUVECs) treated with GOx and iNG128 caused slight morphological changes, primarily manifested as reduced tube width (Figure 4A). iV markedly suppressed tube formation, as reflected by reduced length of treated microtubes. Notably, iVG128 exhibited the most pronounced inhibitory effect, nearly abolishing tube formation and leaving only sparse, disconnected cell clusters. Quantitative analysis revealed that GOx and iNG128 mainly reduced tube area (Figure 4B), which can be attributed to their metabolic disruption abilities. iV further reduced the number of joints (Figure 4C), demonstrating its anti‐angiogenic capability. Importantly, iVG128 induced the most pronounced inhibition of both the tube area and the number of joints, suggesting a synergistic inhibition of angiogenesis.

To more comprehensively evaluate the anti‐angiogenic effects of the material, we further performed HUVEC migration and proliferation assays. To minimize potential confounding from the direct cytotoxicity of GOx on endothelial cells, the medium was replaced with fresh culture medium 4 h after incubation with the formulations, and conditioned medium collected from tumor cells after 24 h treatment was subsequently used to culture HUVECs (Figure 4D). In the scratch‐wound migration assay, the iV and iVG128 groups retained the largest unclosed wound areas at the endpoint (Figure 4E,F); when the migration rate was calculated by normalizing to the wound area at 0 h, it decreased to 5.85% (Figure 4G). Consistently, the CCK‐8 assay showed that iV and iVG128 partially suppressed endothelial proliferation, reducing HUVEC viability to 86.86% of the control (Figure 4H). Together, these findings corroborate the tube formation results and further support the anti‐angiogenic activity of iVG128.

Overall, these results demonstrate that iVG128 effectively induces tumor cell death by disrupting cellular metabolism through glycolytic inhibition and mitochondrial damage, and simultaneously suppressing neovascularization to restrict nutrient supply.

### 2.8 iLAND Improved Tumor Retention and Reshaped Biodistribution

**FIGURE 5:**
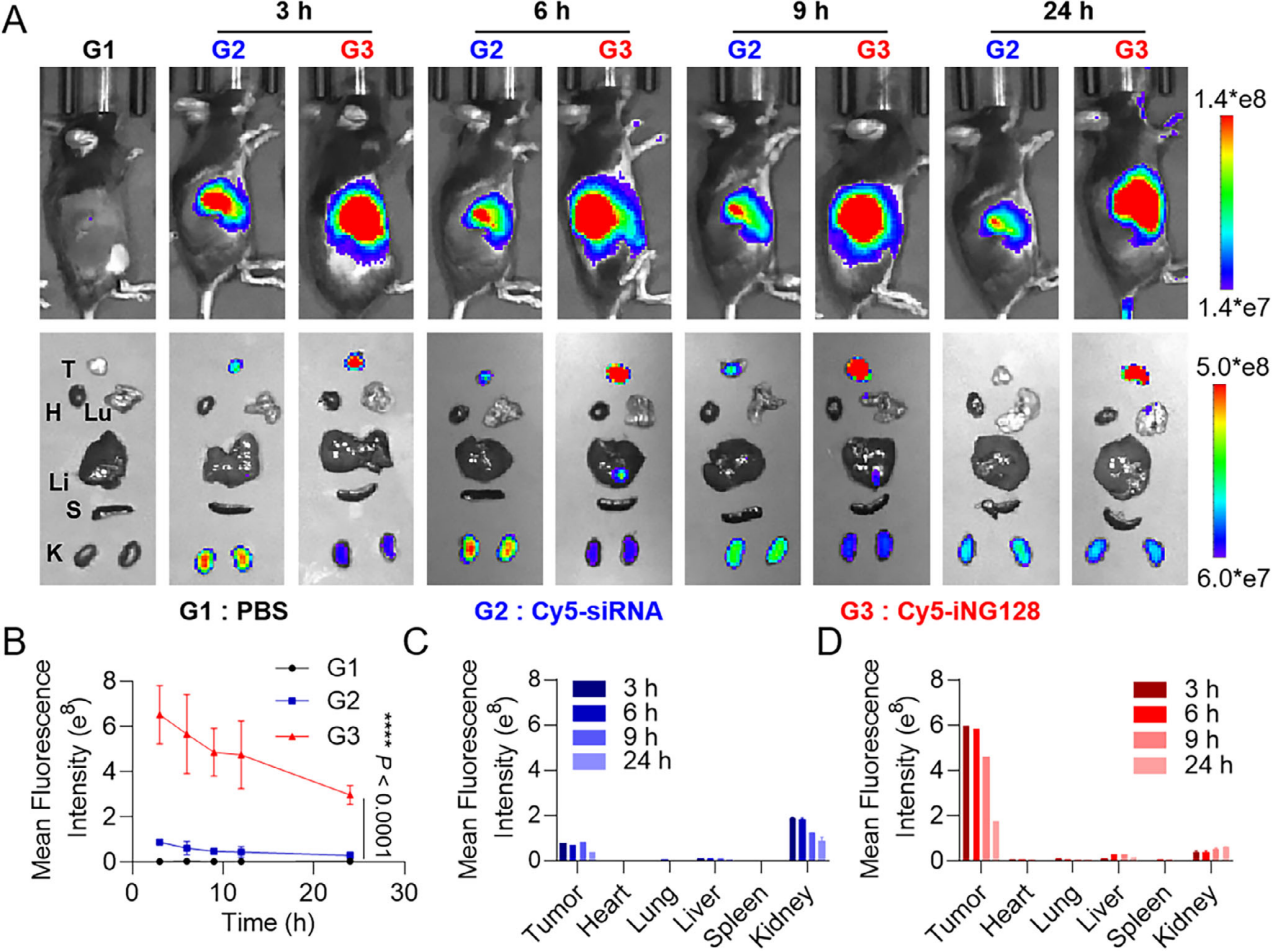
In vivo distribution of iNG128. G1: PBS; G2: Cy5‐siRNA; G3: Cy5‐iNG128. (A) Imaging results of mice and major organs at 3, 6, 9, and 24 h after intratumoral injection. (B) Mean Cy5 fluorescence quantified in mice. (C) Organ imaging of dissected mice at different time points, and quantification of mean fluorescence signals in organs of the Cy5‐siRNA group. (D) Mean fluorescence in organs of the Cy5‐iNG128 group was quantified. Data in B was presented as mean ± S.E.M., with *n* = 2 mice in G1, *n* = 4 mice in G2 and G3. One animal was euthanized at each time point for ex in vivo imaging. *p*‐value < 0.05 was considered statistically significant, with *p < 0.05, **p < 0.01, ***p < 0.001, and ****p < 0.0001.

To assess tumor retention, a tumor‐bearing mouse model was established. When tumor volumes reached approximately 200 mm^3^, mice received intratumoral injections of PBS (Group 1, G1), free Cy5‐siNC (Group 2, G2), or Cy5‐iNG128 (Group 3, G3). Fluorescence signals were recorded at 3, 6, 9, and 24 h after administration. In parallel, one mouse per group was sacrificed for ex vivo imaging of major organs to further analyze biodistribution. Imaging results showed that the fluorescence intensities of Cy5‐iNG128 in tumors were significantly higher than those of Cy5‐siNC within 24 h (Figure 5A). Quantitative analysis (Figure 5B) showed that the difference between G3 and G2 increased from 6.2‐fold at 3 h to 9.7‐fold at 24 h, demonstrating that iLAND substantially prolonged and enhanced siRNA accumulation within tumors.

In addition, we noticed that iLAND substantially changed the metabolic behavior of siRNA. Free siRNA primarily entered systemic circulation, resulting in strong renal fluorescence and minimal tumor accumulation (Figure 5A,C), suggesting rapid renal clearance. Weak liver signals suggested predominant clearance via the kidney–bladder–urine route. By contrast, Cy5‐iNG128 exhibited moderate signals in both kidney and gallbladder (Figure 5D), implying altered pharmacokinetics and partial clearance through the hepatobiliary pathway. Collectively, these findings indicate that iLAND improves intratumoral retention and reshapes the pharmacokinetic profile of encapsulated RNA.

### 2.9 iVG128 Suppressed Tumor Growth in PDX Liver Cancer Model

**FIGURE 6:**
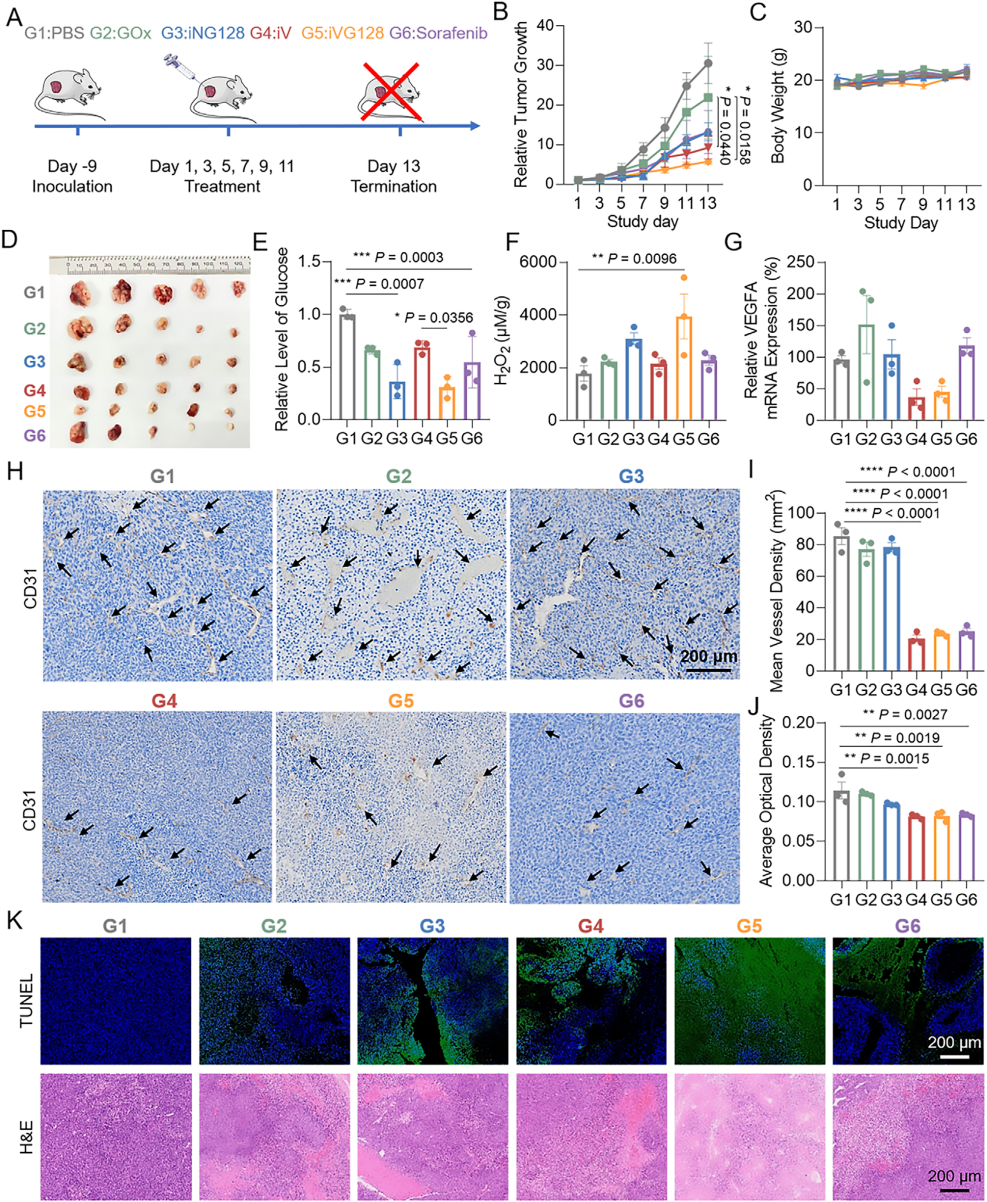
In vivo evaluation of the therapeutic efficacy of iVG128 in a PDX model. (A) Schematic overview of tumor therapy schedule and experimental groups. G1: PBS; G2: GOx; G3: iNG128; G4: iV; G5: iVG128; G6: Sorafenib. (B) Growth curve of tumor volumes during treatment. (C) Changes in body weights of mice during treatment. (D) Images of subcutaneous tumors in mice from different groups after 13 days of treatment. (E) Variation of glucose levels in tumor tissues. (F) Variation of H_2_O_2_ levels in tumor tissues. (G) Inhibition of VEGF gene expression. (H) Immunohistochemical analysis of CD31. Scale bar: 100 µm. (I) Quantification of average microvessel density in tumor sections. (J) Quantification of average optical density of CD31‐positive areas in tumor sections. (K) TUNEL and H&E staining of tumor tissues from G1–G6 groups. Scale bar: 200 µm. Quantitative data are presented as mean ± S.E.M., with *n* = 5 mice per group for (B–D) and *n* = 3 mice per group for (E–K). *p*‐value < 0.05 was considered statistically significant, with *p < 0.05, **p < 0.01, ***p < 0.001, and ****p < 0.0001.

To investigate the therapeutic efficacy of iVG128 in a clinically relevant setting, a patient‐derived xenograft (PDX) liver cancer model was established in BALB/c‐nu mice, aiming to closely replicate the characteristics of naturally occurring human liver cancer. The animals were randomly assigned into six distinct treatment groups when the tumor volumes were around 50 mm^3^: (1) PBS (2) GOx (3) iNG128 (4) iV (5) iVG128 (6) Sorafenib, a clinically approved VEGFR‐targeting agent [27] (Figure 6A). It should be noted that Sorafenib was included in this study as a clinically relevant reference drug. As expected, Sorafenib significantly curtailed tumor growth (Figure 6B and Figure S5), with tumors exhibiting a 12.2‐fold volume increase compared to a 29.5‐fold increase in the PBS group. Free GOx produced a moderate effect (20.9‐fold increase), which was enhanced by iLAND delivery (12.2‐fold increase). Unlike in vitro experiments, iV significantly inhibited tumor growth, underscoring the contribution of angiogenesis suppression in vivo. Strikingly, iVG128 achieved the most potent therapeutic effect, limiting tumor growth to a 4.7‐fold increase.

Body weight monitoring revealed no significant differences across groups, indicating good systemic tolerability (Figure 6C). At two days post‐final treatment, tumors were excised for further analysis. Macroscopic examination showed that iVG128 induced uniform and profound tumor suppression (Figure 6D). Intratumoral glucose levels were markedly reduced in the iVG128 group (Figure 6E). Measurement of intratumoral H_2_O_2_ revealed markedly elevated levels in iNG128‐ and iVG128‐treated tumors, but not in those treated with free GOx (Figure 6F), suggesting that LNP encapsulation promotes cellular internalization of GOx. Quantitative polymerase chain reaction (qPCR) confirmed effective VEGFA silencing in iV‐ and iVG128‐treated tumors (Figure 6G). Immunofluorescence staining of CD31 demonstrated significant suppression of angiogenesis in iV‐, iVG128‐, and Sorafenib‐treated groups, whereas those formulations without siVEGFA (G1–G3) showed no appreciable vascular changes (Figure 6H). Quantitative analysis demonstrated siVEGFA reduced both the number of blood vessels (Figure 6I) and microvessel density (Figure 6J) in tumor tissue. Terminal deoxynucleotidyl transferase (TdT)‐ mediated dUTP nick end labeling (TUNEL) assay and hematoxylin and eosin (H&E) staining revealed increased apoptosis and histological disruption across all therapeutic treatments (Figure 6K). In addition, H&E staining of major organs (Figure S6) showed no obvious pathological abnormalities. No off‐target VEGFA silencing was detected in the liver (Figure S7). Importantly, to directly assess potential systemic oxidative stress arising from GOx‐catalyzed H_2_O_2_ generation after repeated dosing, we quantified H_2_O_2_ levels in major organs (heart, liver, spleen, lung, and kidney) (Figure S8). H_2_O_2_ levels in these organs showed no significant differences compared with the PBS group and no evidence of organ‐specific H_2_O_2_ accumulation.

### 2.10 iVG128 Reverses siVEGFA‐Induced Metabolic Reprogramming

**FIGURE 7:**
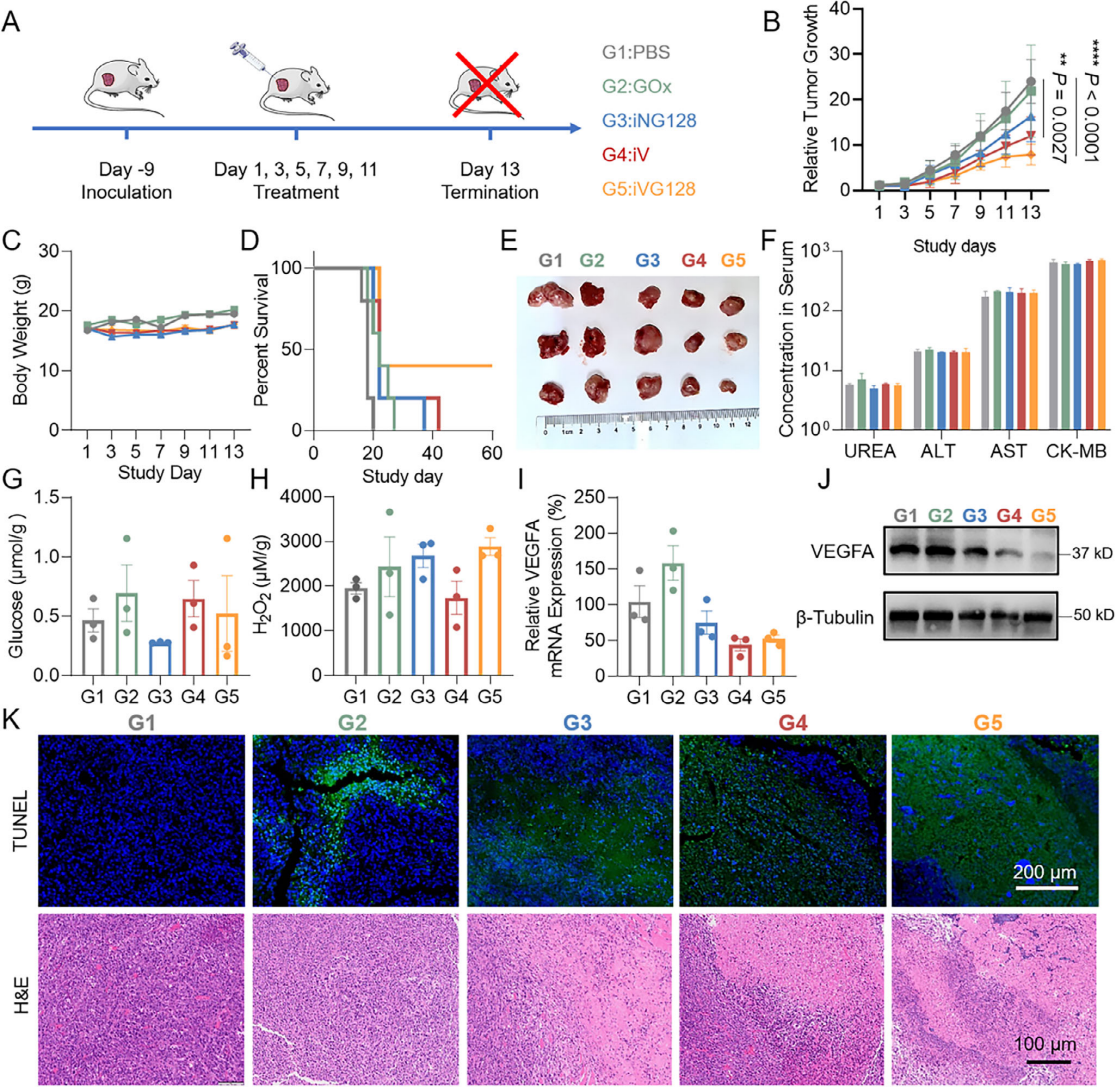
Therapeutic efficacy of iVG128 in colorectal cancer. (A) Schematic overview of tumor therapy schedule and experimental groups. G1: PBS; G2: GOx; G3: iNG128; G4: iV; G5: iVG128. (B) Growth curve of tumor volumes during treatment. (C) Body weight changes of mice throughout the treatment. (D) Survival analysis of tumor‐bearing mice. (E) Images of subcutaneous tumors in mice from different groups after 13 days of treatment. (F) Results of serum biochemical analysis. (G, H) Changes in glucose levels and H_2_O_2_ levels in tumor tissues. (I) Inhibition of VEGF gene expression. (J) Expression of VEGF protein in tumor tissues. (K) TUNEL and H&E staining of tumor tissues from G1–G5 groups. Scale bar: 100 µm. Quantitative data are presented as mean ± S.E.M., with *n* = 8 mice per group for (B, C), *n* = 5 mice per group for D and *n* = 3 mice per group for (E–K). *p*‐value < 0.05 was considered statistically significant, with *p < 0.05, **p < 0.01, ***p < 0.001, and ****p < 0.0001.

Consequently, to explore the underlying mechanisms of the efficient anti‐tumor effects of iVG128, we conducted another in vivo test using a CT26 cell line‐derived xenograft (CDX) subcutaneous tumor model (Figure 7A). Consistent with our prior findings, iVG128 exerted the strongest tumor growth inhibition (Figure 7B and Figure S9). Body weight remained stable across all groups (Figure 7C), indicating excellent tolerability. Survival analysis further supported the therapeutic benefit of iVG128. All PBS‐treated mice died by Day 20. Treatment with GOx, iNG128, or iV extended survival to Day 27, 37, and 42, respectively, whereas two mice in the iVG128 group achieved complete tumor regression and survived until study termination (Figure 7D). After completing the treatment, three animals from each group were sacrificed for comprehensive evaluation. Gross tumor morphology (Figure 7E) visually confirmed the tumor growth inhibition achieved with all active formulations. Serum biochemical analyses indicated no significant systemic safety concerns (Figure 7F). In addition, cytokine profiling revealed no detectable systemic immune activation after repeated administration, as evidenced by comparable levels of representative pro‐inflammatory cytokines (IL‐6, TNF‐α, and IL‐1β) and anti‐inflammatory/immunoregulatory cytokines (IL‐4 and IL‐10) among different groups (Figure S10). Consistently, compared with the PBS group, H_2_O_2_ levels in major organs and VEGFA expression in the liver showed no significant changes (Figures S11 and S12). Furthermore, the functional evaluations of GOx and siVEGFA were consistent with their inherent tumor‐suppressive activities (Figure 7G–J). TUNEL and H&E staining confirmed effective apoptosis and histopathological changes in tumor tissues (Figure 7K), with negligible toxicity to major organs (Figure S13).

To elucidate the metabolic mechanisms underlying the therapeutic effects, tumor tissues were subjected to untargeted metabolomics analysis. This comprehensive approach allowed us to detect a total of 18,817 metabolic features, from which we identified 1,981 unique metabolites after integrating data from both positive and negative ion modes (Figure S14). Volcano plots (Figure S15) revealed that iNG128 mediated 142 upregulated and 72 downregulated metabolites, whereas iV induced broader metabolic perturbations, with 468 upregulated and 97 downregulated metabolites. iVG128 exerted the most pronounced impact, altering 977 metabolites upward and 264 downward, underscoring its potent effect on tumor metabolic reprogramming. Consistent trends were observed in the negative ion mode (Figure S16).

**FIGURE 8:**
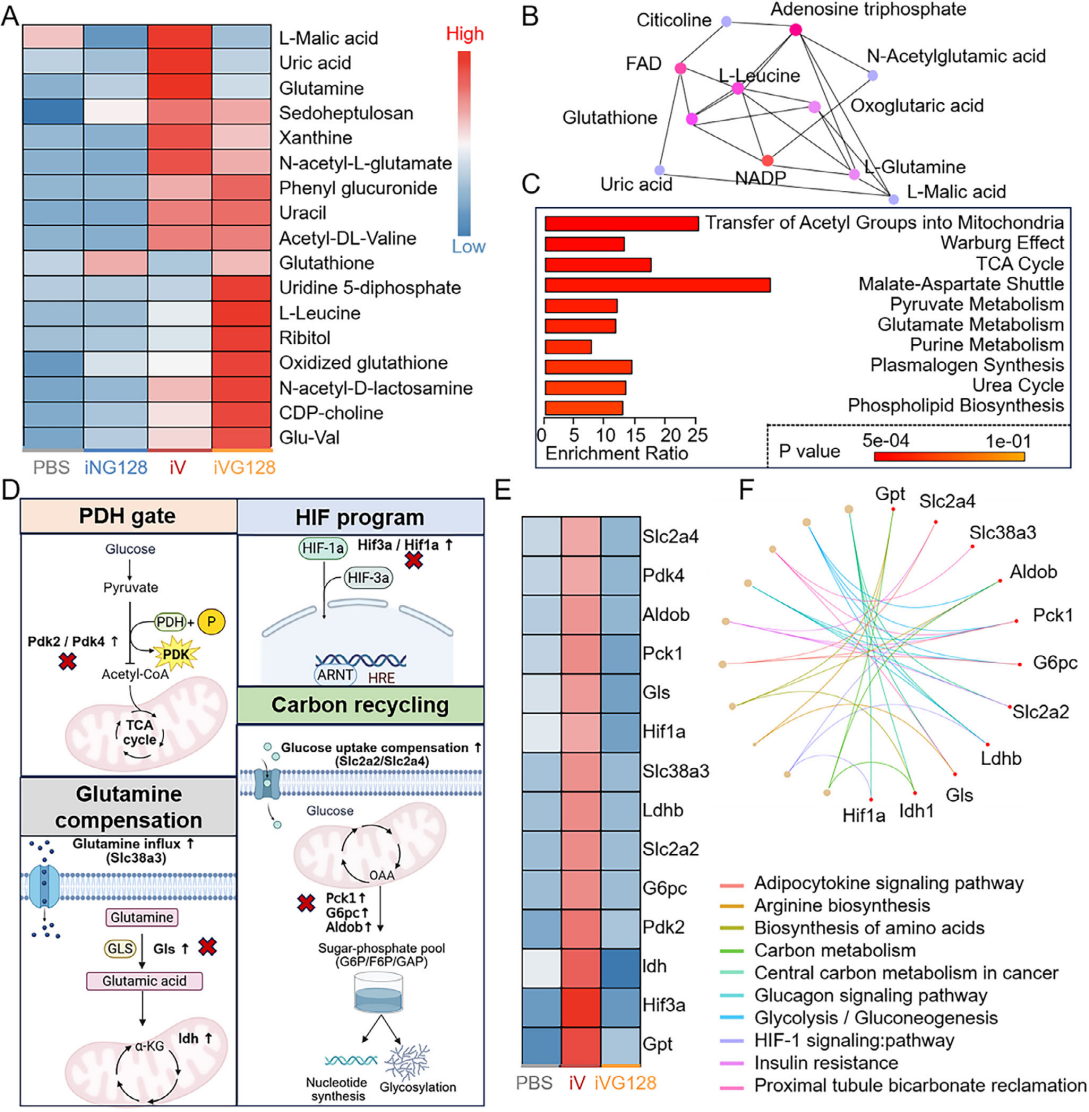
Metabolomic profiling of differential metabolites and pathway enrichment analysis. (A) The heatmap of differential metabolites. (B) Network map of the interactions among differential metabolites. Red colors indicate involvement in a greater number of metabolic pathways. (C) KEGG enrichment bar chart of metabolic pathways. Each bar represents one enriched metabolic pathway. The length of the bar indicates the enrichment ratio, with longer bars representing higher enrichment. (D) Schematic of iV‐induced metabolic reprogramming and its reversal by iVG128 toward the PBS baseline. iV activates coordinated modules involving hypoxia signaling (*Hif3a*/*Hif1a*), PDH gate inhibition (*Pdk2*/*Pdk4*), glutamine compensation (*Slc38a3*, *Gls*/*Idh*), and gluconeogenesis‐like carbon recycling with glucose uptake compensation (*Pck1*/*G6pc*/*Aldob*, *Slc2a2*/*Slc2a4*) to support the sugar‐phosphate pool and biosynthesis. These adaptive programs are broadly blunted in iVG128, showing a reversal toward PBS. (E) Heatmap of selected metabolism‐related genes derived from RNA‐seq across PBS, iV, and iVG128 groups. (F) Gene–pathway bipartite network highlights convergence of adaptive rewiring genes on central carbon metabolism and hypoxia‐associated pathways. Bipartite network linking selected genes (red nodes, right) to enriched KEGG pathways (beige nodes, left). Edges indicate pathway annotation membership; edge colors denote pathway categories. Pathway node size reflects the number of genes (Count) mapped to each pathway within the selected set. The network shows prominent convergence on glycolysis/gluconeogenesis, central carbon metabolism (including cancer‐associated carbon metabolism), amino acid biosynthesis, and HIF‐1 signaling. All quantitative results are presented as the mean ± S.E.M. (*n* = 3). *p*‐value < 0.05 was considered statistically significant, with *p < 0.05, **p < 0.01, ***p < 0.001, and ****p < 0.0001.

Orthogonal partial least squares discriminant analysis (OPLS‐DA) identified the key differential metabolites, visualized in a heatmap (Figure 8A). iV treatment reduced glucose availability via angiogenesis inhibition, triggering compensatory pathways such as glutamine metabolism and the pentose phosphate pathway (PPP). Elevations in glutamine, L‐malic acid and sedoheptulosan reflect this adaptive response, which replenishes TCA cycle intermediates and supports nucleotide biosynthesis. Such a reprogramming pattern aligns with adaptive resistance to anti‐VEGF drugs. Elevations in nucleotide and amino acid catabolic products (uric acid, xanthine, uracil, acetyl‐DL‐valine) further indicated metabolic adaptation under nutrient stress. In contrast, iNG128 showed only limited metabolic impact.

Strikingly, iVG128 nearly normalized L‐malic acid, glutamine, and uric acid levels, suggesting that compensatory TCA flux was nearly abolished. Concurrently, the levels of uridine diphosphate (UDP), L‐leucine, ribitol, oxidized glutathione (GSSG), N‐acetyl‐D‐lactosamine, cytidine diphosphate (CDP)‐choline, and glutamyl‐valine (Glu‐Val) were increased, indicating the activation of secondary adaptive pathways involving nucleotide, amino acid, and phospholipid metabolism. Consistently, a focused nicotinamide adenine dinucleotide phosphate (reduced form, NADPH)‐balance heatmap (Figure S17) showed an iV‐associated PPP/pentose signature (e.g., 6‐phospho‐D‐gluconate and pentose phosphates), whereas iVG128 attenuated this PPP‐linked profile while elevating ribitol and GSSG. This pattern supports a remodeling of NADPH/redox homeostasis during the transition from primary compensation (iV) to secondary adaptation (iVG128).

We then compared iVG128‐ and iNG128‐treated tumors. Network analysis (Figure 8B) discovered that the NADP played a central role in the altered network, emphasizing its impact on cellular metabolism. The KEGG enrichment (Figure 8C) highlighted mitochondrial pathways, including the TCA cycle, malate‐aspartate shuttle, and acetyl group transport, alongside suppression of Warburg‐related metabolism. These results indicate that siVEGFA drives primary metabolic compensation, whereas combination with GOx significantly reversed metabolic reprogramming, thereby enhancing metabolic interference following anti‐VEGF therapy.

### 2.11 iVG128 Blunts the Hypoxia PDH Gate and Glutamine Compensation Axis at the Transcriptional Level

To further elucidate the molecular basis by which iVG128 reverses siVEGFA (iV)‐induced metabolic reprogramming, we performed transcriptomic sequencing of tumor tissues and compared differentially expressed genes (DEGs) between the iV and iVG128 groups (adjusted *p*‐value, *p*_adj_ < 0.05). Most significant DEGs were upregulated in the iV group but broadly declined in iVG128, indicating that iVG128 blunts the adaptive transcriptional response triggered by iV (Figure S18). We then focused on metabolism‐related genes and their associated regulatory pathways. The transcriptional programs impacted by iV spanned hypoxia response, control of pyruvate entry (the PDH gate), glutamine‐centered compensation, and gluconeogenesis‐like carbon recycling; notably, these coordinated modules were collectively shifted downward toward the PBS baseline in the iVG128 group (Figure 8D).

Specifically, iV markedly increased *Hif3a*/*Hif1a*, consistent with activation of a HIF‐driven hypoxia/stress program. Induction of *Pdk2*/*Pdk4* pointed to PDH gate remodeling, which is expected to enhance inhibitory phosphorylation of the PDH complex, thereby restricting pyruvate entry into mitochondria and reducing the contribution of glucose‐derived carbon to the TCA cycle. Against this backdrop, iV further engaged a glutamine compensation network. Upregulation of *Slc38a3* (a system N neutral amino acid transporter with preference for glutamine) suggested enhanced glutamine influx, while increased *Gls* indicated elevated potential for glutamine‐to‐glutamate conversion. The concurrent elevation of *Gpt* and *Idh* supported activation of C–N exchange and recruitment of oxidative metabolic nodes, enabling replenishment of the TCA cycle via α‐ketoglutarate and related intermediates to sustain bioenergetic and biosynthetic demands. In addition, coordinated upregulation of *Pck1*, *G6pc*, and *Aldob* indicated engagement of a gluconeogenesis‐like/carbon recycling signature; together with increased glucose transporters (*Slc2a2*/*Slc2a4*) and the lactate‐axis component *Ldhb*, these patterns suggest that iV promotes substrate acquisition and carbon rerouting to maintain the sugar‐phosphate intermediate pool and to tune glycolysis‐associated outputs under vascular supply–limited metabolic stress.

Importantly, iVG128 broadly suppressed the above transcriptional modules and shifted them back toward baseline (Figure 8E), indicating that GOx co‐delivery is not merely an additional stressor layered onto iV. Rather, sustained glucose depletion coupled with mitochondrial metabolic stress likely limits the persistence of the iV‐triggered adaptive flux reallocation at the transcriptional level, providing direct molecular support for iVG128‐mediated reversal of metabolic reprogramming. Consistently, the gene–pathway (KEGG) bipartite network showed that these genes converge on core pathways including HIF signaling, glycolysis/gluconeogenesis, and central carbon metabolism, supporting their pathway‐level integration as a “stress–flux reallocation” compensatory network (Figure 8F).

## 3 Discussion and Conclusion

Anti‐angiogenic therapy can effectively restrict vasculature‐mediated nutrient supply; however, its long‐term efficacy is often limited by the intrinsic metabolic plasticity of tumors. In this study, we take the “metabolic compensation” that emerges after VEGF inhibition as a key entry point and propose a combinatorial intervention to counter therapeutic escape from VEGF inhibitors. Unlike prior approaches that used glucose oxidase (GOx) merely as a generic “starvation/sensitization module” to be simply added to chemotherapy or photothermal therapy [28, 29, 30], we emphasize the potential of GOx to reverse VEGF inhibition‐induced compensatory reprogramming by targeting metabolic pathways. To this end, we employed the iLAND ionizable lipid nanoplatform for co‐delivery of siVEGFA and GOx and, through systematic formulation screening, identified iVG128 as the optimized lead formulation. iVG128 not only exhibits favorable physicochemical stability and biocompatibility, but also enhances tumor retention while preserving the catalytic activity of GOx.

Mechanistically, multi‐omics evidence indicates that the efficacy of iVG128 arises from “deep mechanistic synergy” rather than simple additive toxicity. We observed that siVEGFA alone (iV), while suppressing angiogenesis, elicited a canonical compensatory metabolic response, including accumulation of TCA cycle–related metabolites (e.g., glutamine and L‐malic acid) and upregulation of hypoxia‐adaptive transcriptional programs and PDH gate remodeling genes (e.g., *Pdk2*/*Pdk4*). These findings suggest that under glucose‐restricted conditions, tumors tend to rely on alternative substrates such as glutamine to sustain oxidative metabolism and biosynthetic demands. In sharp contrast, iVG128 markedly reversed these compensatory features and broadly suppressed the corresponding adaptive programs at the transcriptional level. Together with evidence of GOx‐induced oxidative stress and mitochondrial ultrastructural damage, we propose that iVG128 establishes a synergistic closed loop: siVEGFA blocks external nutrient supply (“source reduction”), whereas GOx not only limits glycolysis by consuming glucose but also impairs the mitochondrial oxidative capacity required for compensation via alternative fuels (e.g., glutamine) (“flow restriction”). This dual strike ultimately drives a systemic collapse of the tumor metabolic network. These properties collectively ensure potent antitumor efficacy across multiple models, outperforming both monotherapies and the clinical standard Sorafenib. Notably, Sorafenib was included only as a clinically relevant benchmark rather than a direct pharmacological comparator. To intuitively evaluate the antitumor efficacy of iVG128, both agents were administered under their respective optimal preclinical dosing regimens. This comparison strategy has been widely accepted both in preclinical and clinical practice to demonstrate the therapeutic superiority of a novel agent [31, 32, 33, 34]. Imporantly, because GOx catalysis depends on local glucose and oxygen availability, differences in tumor perfusion and hypoxia across tumor types may cause variability in enzymatic activity and therapeutic response; therefore, further optimization of the siRNA/GOx ratio and dosing regimen may be required when extending this strategy to additional models. Meanwhile, whether the in vivo release/activation kinetics of the two cargos are synchronized remains to be further validated.

In summary, this study establishes a proof‐of‐concept for a combinatorial metabolic and anti‐angiogenic therapeutic strategy using the multifunctional nanoplatform iVG128. By simultaneously targeting aerobic and anaerobic glucose utilization and VEGFA‐driven angiogenesis, iVG128 not only suppresses vascular‐mediated nutrient supply but also induces tumor cell death through energetic collapse and mitochondrial dysfunction. Beyond its potent antitumor efficacy, iVG128 demonstrates favorable stability, biocompatibility, and tumor retention. Collectively, this work underscores the value of integrating metabolic interference with anti‐angiogenic therapy, providing a versatile platform for the rational design of nanotherapeutics to treat cancer by depleting the energy supply.

## 4 Materials and Methods

### 4.1 Materials

GOx was obtained from Sangon Biotech (China). The small interfering RNA targeting VEGFA and firefly luciferase were ordered from GenePharma (China). Cholesterol, 1,2‐distearoyl‐sn‐glycero‐3‐phosphocholine (DSPC) and 1,2‐dimyristoyl‐rac‐glycero‐3‐methoxypolyethylene glycol‐2000 (DMG‐PEG_2000_) were purchased from A.V.T. Pharmaceutical Co., Ltd. (Shanghai, China). Fetal bovine serum (FBS) was purchased from PAN Biotech (Germany). Penicillin−streptomycin, Lipofectamine 2000, 1640 medium, Dulbecco's modified Eagle's medium (DMEM), Opti‐MEM, LysoTracker, Quant‐iT RiboGreen RNA Assay kit and trypsin were purchased from Thermo Fisher Scientific Inc., (USA). DAPI and Hoechst 33342 were purchased from Beyotime Biotechnology (China). YeaRed Nucleic Acid Gel Stain (10202ES76), Annexin V‐FITC/PI Apoptosis Assay Kit (40302ES60), TUNEL Apoptosis Detection Kit (FITC) (40306ES20) and Hieff qPCR SYBR Green Mix (11201ES03) were purchased from YEASEN (China). FreeZol Reagent (R711‐01) and Hiscript III Reverse Transcriptase (R302‐01) were purchased from Vazyme Biotech (China). Anti‐VEGFA antibody was purchased from Abcam (UK). BCA protein concentration determination kit (CW2011) was purchased from CWBIO (China). Nitrocellulose membrane was purchased from Millipore (USA). Prestained Protein Ladder 10–180 kDa was purchased from Genetech (China). Lactic Acid (LA) Content Assay Kit (BC2235) was purchased from Solarbio Life Science (China). Enhanced ATP Assay Kit (S0027), H_2_O_2_ Assay Kit (S0038), Mitochondrial Membrane Potential Assay Kit (C2006) and Reactive Oxygen Species (ROS) Assay Kit (S0033S) were purchased from Beyotime (China). Glucose Assay Reagent (J6021) was purchased from Promega (USA).

### 4.2 Animals

BALB/c and BALB/c‐nu mice were obtained from SPF (Beijing) Biotechnology Co., Ltd. (Beijing, China). Mice were maintained in a temperature‐controlled, ventilated facility under a 12 h light/12 h dark cycle, with free access to food and water. All procedures were approved by the Ethics Committee of Beijing Institute of Technology and performed following the Guide for the Care and Use of Laboratory Animals (License No: BIT‐EC‐SCXK(Beijing)20190010‐M‐2021010).

### 4.3 The Preparation of iLAND

iLAND was prepared as described previously [24]. Briefly, ionizable lipid A1‐D1‐5, DSPC, cholesterol, and DMG‐PEG2000 were each separately dissolved in ethanol at a final concentration of 20 mg mL^−1^. The lipid components were then mixed in a molar ratio of A1‐D1‐5: DSPC: cholesterol: DMG‐PEG2000 = 54:12:60:2.5. Subsequently, the lipid mixture was rapidly injected into three times the volume of sodium citrate buffer (50 mm, pH 4.0) to form iLAND nanoparticles.

### 4.4 GOx and siRNA Encapsulation

GOx was dissolved in water at a concentration of 5 mg mL^−1^. siRNA was dissolved in 25% ethanol at a concentration of 500 ng µL^−1^. iLAND and siRNA were mixed at a mass ratio of 15:1 based on previous screening results. Additionally, GOx was added to iLAND at varying mass ratios, ranging from 8:1 to 256:1. The resulting formulation was incubated at 50°C for 20 min and then dialyzed against 1× PBS for at least 2 h.

### 4.5 GOx Enzyme Activity Determination

To release encapsulated GOx, an equal volume of 2% (w/w) Triton X‐100 was added and incubated at room temperature for 30 min. Free GOx mixed with Triton X‐100 served as a control. Enzymatic activity was assessed by incubating GOx with glucose using the protocol of the manufacturer. The pH of the reaction solution was measured every 15 min to monitor changes over time.

### 4.6 Physicochemical Characterization of LNPs

Particle size and zeta potential were characterized by dynamic light scattering (Zetasizer 3000HS, Malvern Instruments) at a wavelength of 677 nm and a scattering angle of 173° at room temperature. Morphology was examined by TEM. The concentrations of GOx and siRNA in lipid nanoparticles (LNPs) were quantified using the BCA protein assay and RiboGreen RNA assay, respectively, according to the manufacturers' protocols.

### 4.7 Intracellular H_2_O_2_ Detection

CT26 cells were seeded into 6‐well plates at 2×10^5^ cells per well and cultured in DMEM supplemented with 1 mg mL^−1^ glucose. After 24 h, GOx or iNG128 (0.022 mg mL^−1^) was added and incubated for 12 h. Cells were then collected, and lysis buffer was added at a volume of 100 µL per 1×10^6^ cells. Following centrifugation at 12 000 *g* and 4 °C for 5 min, the supernatant was placed into a 96‐well plate. Reaction reagents were added, and fluorescence intensity was measured at 560 nm following a 30 min incubation at room temperature. H_2_O_2_ concentrations were calculated based on a standard curve.

### 4.8 pKa Determination

The pKa of iLAND and iNG128 was determined using 2‐(p‐toluidino)‐6‐naphthalene sulfonic acid (TNS) as a pH‐sensitive probe, following a previously published protocol [35]. Briefly, nanoparticles (100 µm) were prepared in a buffer composed of 10 mm ammonium acetate, 10 mm HEPES, 10 mm MES, and 130 mm NaCl. The pH was varied between 2.5 and 11, followed by addition of 1 µm TNS. Fluorescence measurements were taken at 445 nm, and the pKa was calculated as the pH yielding half‐maximal fluorescence.

### 4.9 Cell Viability Test

CT26 cells were inoculated into 96‐well plates at 1×10^4^ cells per well and maintained overnight prior to transfection. Six formulations with varying iLAND:GOx mass ratios (8:1 to 256:1) and a fixed iLAND:siRNA mass ratio (15:1) were prepared. On the following day, samples were added at a final siRNA concentration of 100 nm and incubated for 12 h. Afterward, the culture medium was replaced with fresh medium containing 10% (v/v) CCK‐8 and incubated for 1 h. Absorbance at 450 nm was recorded using a microplate reader.

### 4.10 Cell Apoptosis and Cell Cycle Analysis

CT26 cells were seeded in 6‐well plates at a density of 2×10^5^ cells per well and cultured for 24 h. Nanoparticles were added to achieve a final siRNA concentration of 100 nm, with free GOx added to the control wells at concentrations equivalent to those in iNG128 and iVG128 formulations. After 12 h of incubation, cells were harvested and washed three times with PBS and stained with Annexin V‐FITC and PI for 10 min at room temperature to detect apoptosis. To analyze the cell cycle, cells were fixed in 70% ethanol at 4 °C for 2 h, washed, and stained with PI and RNase A. Fluorescence was analyzed by flow cytometry (FACS), and data were processed using FlowJo software (v10).

### 4.11 Measurement of Gene and Protein Expression

The procedures for cell culture and formulation transfection were as described above. To assess relative gene expression, cells were transfected for 24 h. Afterward, the medium was replaced with FreeZol Reagent (1 mL per well) to lyse the cells. The lysates were collected and mixed with chloroform (200 µL), then incubated for 3 min. After centrifugation at 12,000 rpm for 10 min, the aqueous fraction was carefully collected into clean tubes, combined with an equal amount of isopropanol, and incubated for 10 min. Following a second centrifugation, total RNA was precipitated and collected. One microgram of RNA was reverse transcribed into complementary DNA (cDNA) using the protocol of the manufacturer. Gene silencing efficiency of siVEGFA was evaluated via qPCR, and VEGFA expression was normalized to β‐actin.

For protein expression analysis, cells were lysed in passive lysis buffer supplemented with protease inhibitors. After complete lysis, samples were centrifuged at 12,000 rpm for 10 min, and the supernatant was collected and kept on ice. Protein content was quantified using a BCA Protein Assay Kit. Samples containing 20 µg of protein were mixed with 5× loading buffer and denatured at 99 °C for 10 min. Samples, along with a protein ladder, were separated on 10% SDS‐PAGE gels and transferred to nitrocellulose membranes. Membranes were blocked with 5% BSA for 1 h, then incubated with primary antibodies overnight at 4 °C. After washing with TBST, membranes were incubated with HRP‐conjugated secondary antibodies for 1 h at 4 °C. Protein bands were visualized using a 5200 Multi‐Automated Chemiluminescence System (Tanon, China).

### 4.12 Exploration of Internalization Mechanisms

CT26 cells were incubated for 30 min with methyl‐β‐cyclodextrin (5 mm), genistein (1 mm), amiloride‐HCl (100 µm), chlorpromazine‐HCl (30 µm), or culture medium as a control. Cy5‐labeled iNG128 was then added to each group at a final siRNA concentration of 100 nm. Cells were incubated at either 37 °C or 4 °C for 4 h. After incubation, cells were harvested from 12‐well plates using 0.25% trypsin, washed with PBS, and analyzed by flow cytometry (FACSAria II, BD, USA). Data were processed using FlowJo 10 software.

### 4.13 Subcellular Localization Observation

CT26 cells were seeded in 35 mm dishes one day prior to transfection. Adherent cells were transfected with Cy5‐iNG128. At 0, 1, 3, 5, 8, and 10 h post‐transfection, the medium was removed, and cells were washed with PBS. Cells were then stained with Hoechst 33342 and LysoTracker Green for 15 min. Images were captured using confocal microscopy (N‐SIM A1R, Nikon, Japan). Image analysis was performed using NIS‐Elements Viewer software.

### 4.14 ATP and Lactate Production

Cell culture and formulation transfection were carried out as described above. ATP and lactate production were quantified following the manufacturers' protocols.

### 4.15 Intracellular ROS Measurement

CT26 cells were seeded in 6‐well plates. After attachment, the culture medium was removed, cells were gently washed once with PBS, and replaced with low‐glucose medium. Cells were then treated with the indicated formulations at a final siRNA concentration of 100 nm. The experimental groups included Mock, GOx, iV, iNG128, and iVG128. Intracellular ROS was assessed using the DCFH‐DA probe (1:1000 dilution). Before staining, the culture medium was removed, and 1 mL of DCFH‐DA working solution was added to each well, followed by incubation at 37°C for 20 min. Cells were then washed three times with HBSS, and DCF fluorescence was collected using a fluorescence detection system.

### 4.16 Mitochondrial Membrane Potential Assay

CT26 cells were seeded in 6‐well plates and allowed to attach. The culture medium was removed, cells were gently washed once with PBS, and replaced with low‐glucose medium. Cells were then treated with the indicated formulations at a final siRNA concentration of 100 nm. For JC‐1 staining, the medium was removed, and each well was supplemented with 1 mL fresh medium followed by 1 mL JC‐1 working solution (prepared according to the manufacturer's instructions), mixed thoroughly, and incubated at 37°C for 20 min. After incubation, the supernatant was aspirated and cells were washed twice with pre‐chilled JC‐1 staining buffer. Finally, 2 mL culture medium was added per well, and images were acquired using a confocal laser scanning microscope.

### 4.17 Tube Formation Assay

HUVECs were seeded in 6‐well plates. The formulations (iV, iNG128, and iVG128) were freshly prepared the next day and added to the wells at a final siRNA concentration of 100 nm. Free GOx was added in parallel at an equivalent concentration to that in iNG128 and iVG128. Matrigel (50 µL per well) was added to a pre‐cooled 96‐well plate and incubated at 37 °C. After 4 h, treated cells were trypsinized and seeded onto Matrigel‐coated wells (20,000 cells per well, in quadruplicate). After 14 h of incubation, tube‐like structures were imaged using a microscope (Olympus IX71, Tokyo, Japan). Microtubules with ≥3 junctions were analyzed for length and area using ImageJ.

### 4.18 HUVEC Proliferation Assay

CT26 cells were seeded in 6‐well plates at 2.0×10^5^ cells per well and allowed to attach overnight, followed by treatment with the indicated formulations for 4 h. The medium was then removed, cells were gently washed once with PBS, and replaced with fresh complete medium for an additional 24 h. The supernatant was collected, centrifuged at 300* g* for 5 min to remove debris, and filtered through a 0.22 µm membrane to obtain tumor‐conditioned medium (TCM). HUVECs were seeded in 96‐well plates at 5.0×10^3^ cells per well. After 12 h of attachment, the medium was replaced with a 1:1 mixture of TCM and fresh complete HUVEC medium and incubated for 24 h. Subsequently, CCK‐8 reagent was added and absorbance was measured using a microplate reader to evaluate cell proliferation.

### 4.19 HUVEC Wound‐Healing Assay

HUVECs were seeded in 6‐well plates at 4.0×10^5^ cells per well and cultured for 24 h to form a confluent monolayer. A linear scratch was generated using a 200 µL pipette tip, and detached cells were removed by washing twice with PBS. Cells were then incubated with a 1:1 mixture of serum‐free medium and TCM. Images were acquired at 0 and 48 h to assess cell migration.

### 4.20 In Vivo Biodistribution

Hepa1‐6 cells (1×10^6^ cells per mouse) were subcutaneously injected into C57BL/6 mice. Once tumors reached ≈200 mm^3^, mice were randomly divided into three groups: (1) PBS, (2) free Cy5‐siRNA (1 mg kg^−1^), and (3) Cy5‐iNG128 (1 mg kg^−1^). In vivo fluorescence imaging was performed at 3, 6, 9, and 24 h post‐injection using the IVIS Spectrum CT (PerkinElmer, USA). Afterward, heart, lung, liver, spleen, kidney and tumors were harvested and imaged. Average fluorescence intensity in regions of interest (ROIs) was quantified using LivingImage software.

### 4.21 Anti‐Tumor Efficacy

Two in vivo studies were conducted to evaluate the therapeutic efficacy of iVG128. In the first study, thirty BALB/c‐nu mice bearing patient‐derived xenograft (PDX) tumors were randomly assigned to six groups: (1) PBS, (2) GOx (0.14 mg kg^−1^), (3) iNG128 (GOx = 0.14 mg kg^−1^), (4) iV (1 mg kg^−1^), (5) iVG128 (GOx = 0.14 mg kg^−1^, siVEGFA = 1 mg kg^−1^), (6) Sorafenib (30 mg/kg/day). The mice received treatments every other day for a total of six doses. At two days post‐final dose, the mice were euthanized. Randomly selected three mice for analysis. Blood was obtained for serum biochemical analyses, including UREA, ALT, AST, CK, and LDH, to assess formulation safety. Tumor volume and body weight were measured regularly during the treatment. Major organs and portions of tumors were paraformaldehyde‐fixed for histological studies, while the rest of the tumors were stored frozen in liquid nitrogen.

In the second study, forty BALB/c mice bearing CT26 tumors were divided into five groups and treated as described in Study 1. Two days after the final treatment, three mice from each group were sacrificed, and organs and tumors were harvested. Organs were fixed in 4% paraformaldehyde for histology. Tumor samples were processed for target gene expression analysis, apoptosis detection, H_2_O_2_ levels, and metabolomics. The remaining mice were monitored for survival, tumor progression, and body weight. Mice were euthanized when tumor volumes reached 2,000 mm^3^.

### 4.22 Metabolomics Analysis

Frozen tumor tissues were thawed on ice and homogenized in a cold solvent mixture (methanol: acetonitrile: water, 2:2:1, v/v/v). Following thorough vortexing and 30‐minute low‐temperature sonication, samples were incubated at −20 °C for protein precipitation. After centrifugation (14 000 *g*, 20 min, 4°C), the supernatants were collected and dried under vacuum. Dried metabolites were redissolved in 100 µL of acetonitrile: water (1:1, v/v), vortexed briefly, and clarified by a second centrifugation prior to injection.

Chromatographic separation was carried out using an Agilent 1290 Infinity UHPLC coupled with a HILIC column maintained at 25 °C. The injection volume was 2 µL with a constant flow rate of 0.5 mL min^−1^. Mobile phase A consisted of water with 25 mm ammonium acetate and 25 mm ammonium hydroxide, while mobile phase B was acetonitrile. The gradient began at 95% B for 0–0.5 min, gradually reduced to 65% B over 6.5 min, then to 40% B at 8 min, held briefly, and re‐equilibrated to starting conditions for a total of 12 min.

Mass detection was performed on a TripleTOF 6600 mass spectrometer (AB SCIEX) using electrospray ionization in both positive and negative modes. Raw spectra were converted to mzXML format via ProteoWizard and processed using the XCMS package for peak picking, alignment, and quantification. Identified metabolites were subjected to quality control, followed by downstream analyses including heatmap visualization, network construction, and KEGG pathway enrichment. Data acquisition and processing were completed in collaboration with Shanghai Applied Protein Technology Co., Ltd.

### 4.23 RNA‐Seq

Tumor tissues from different treatment groups were collected for total RNA extraction, followed by cDNA library construction and high‐throughput sequencing. Data acquisition and processing were completed in collaboration with Shanghai Applied Protein Technology Co., Ltd.

### 4.24 Statistical Analysis

All quantitative results are presented as the mean ± standard error of mean (S.E.M.). Two‐group comparisons employed two‐tailed unpaired Student's *t*‐tests, while one‐way ANOVA followed by Tukey's test was used for multiple groups. Analyses were carried out in GraphPad Prism (v8.0.1). A *p*‐value < 0.05 was considered statistically significant, with *p < 0.05, **p < 0.01, ***p < 0.001, and ****p < 0.0001.

## Author Contributions

L.Z. performed most experiments, wrote the manuscript and organized the figures. B.H., S.G., P.W., H.Y., and Y.F. were involved in animal studies. Y.L., Y.X., and Q.L. were involved in data analysis and valuable discussions. B.H. helped L.Z. to perform the project, was involved in detailed discussions, and wrote and revised the manuscript. J.H. provided critical comments for experimental design, and was involved in detailed discussion and data analysis. Y.H. critically revised the manuscript and supervised the entire project.

## Funding

This research was financially supported by the National Natural Science Foundation of China (Grant Nos. 82202338, 32471514 and 32171394), the Fundamental Research Funds for the Central Universities (Grant No. 2022CX01013), the China Postdoctoral Science Foundation (Grant No. 2024M764115) and the National High Level Hospital Clinical Research Funding & Elite Medical Professionals Project of China‐Japan Friendship Hospital (Grant No. ZRJY2023‐ GG04).

## Conflicts of Interest

Y.H. is the founder of Rigerna Therapeutics. The other authors declare no competing interests.

## Supporting information




**Supporting file**: advs75126‐sup‐0001‐SuppMat.docx

## Data Availability

The data that support the findings of this study are available from the corresponding author upon reasonable request.
