# Supplementary material for: Combating VEGFA‐siRNA‐Induced Metabolic Reprogramming via Glucose Utilization Deprivation
Source: Adv Sci (Weinh). 2026 Apr 9;13(36):e19290. doi: 10.1002/advs.202519290 (PMC13317555; doi:10.1002/advs.202519290)
Supplement: Supplementary file 1 — Supporting file: advs75126‐sup‐0001‐SuppMat.docx [file ADVS-13-e19290-s001.docx]

Supporting Information

**Combating VEGFA-siRNA-Induced Metabolic Reprogramming via Glucose Utilization Deprivation**

*Lulu Zheng, Shuai Guo, Yingjixing Luo, Pengfei Wu, Haiyin Yang, Yingqiu Xie, Yuchuan Fan, Qing Liu, Bo Hu^*^, Jia Huang^*^, Yuanyu Huang**^*^*


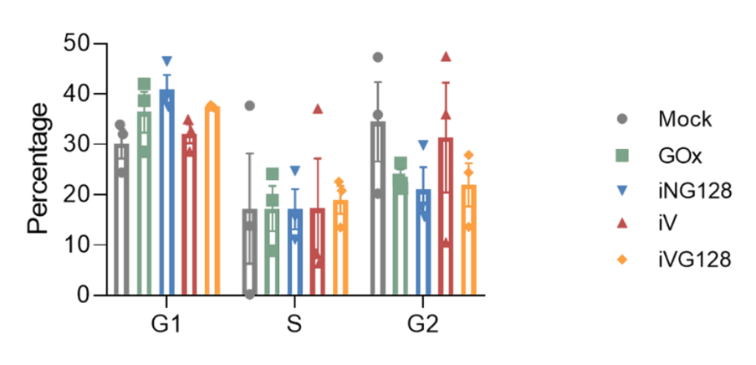


Figure S1. Quantification of cell cycle analysis.


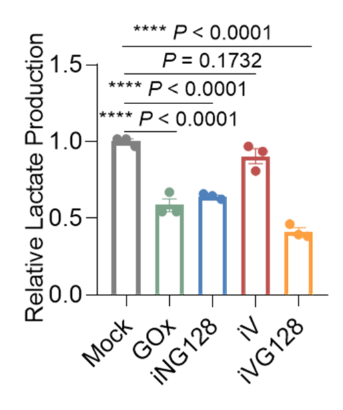


Figure S2. Intracellular lactate concentrations in CT26 cells.


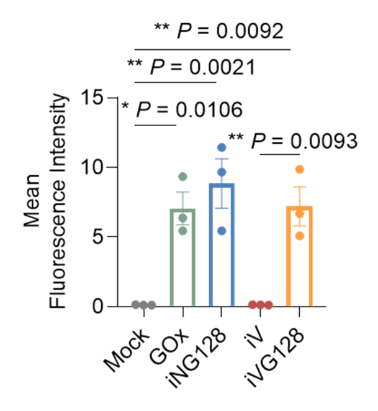


Figure S3. Quantitative analysis of intracellular ROS in CT26 cells after different treatments.


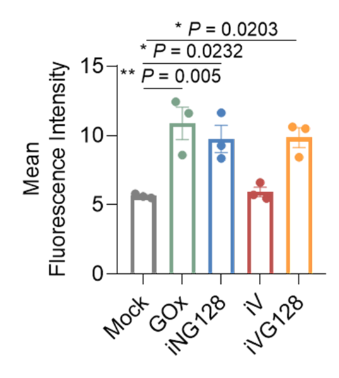


Figure S4. Quantitative analysis of mitochondrial membrane potential (ΔΨm) in CT26 cells after different treatments.


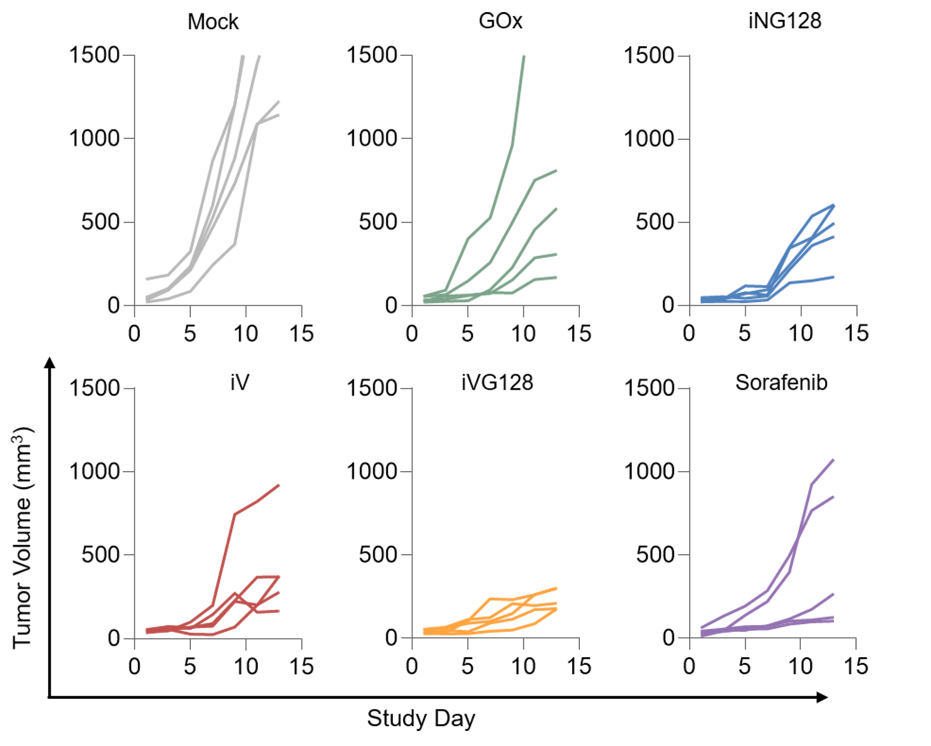


Figure S5. Tumor growth curves of mice from different groups during treatment in the PDX model.


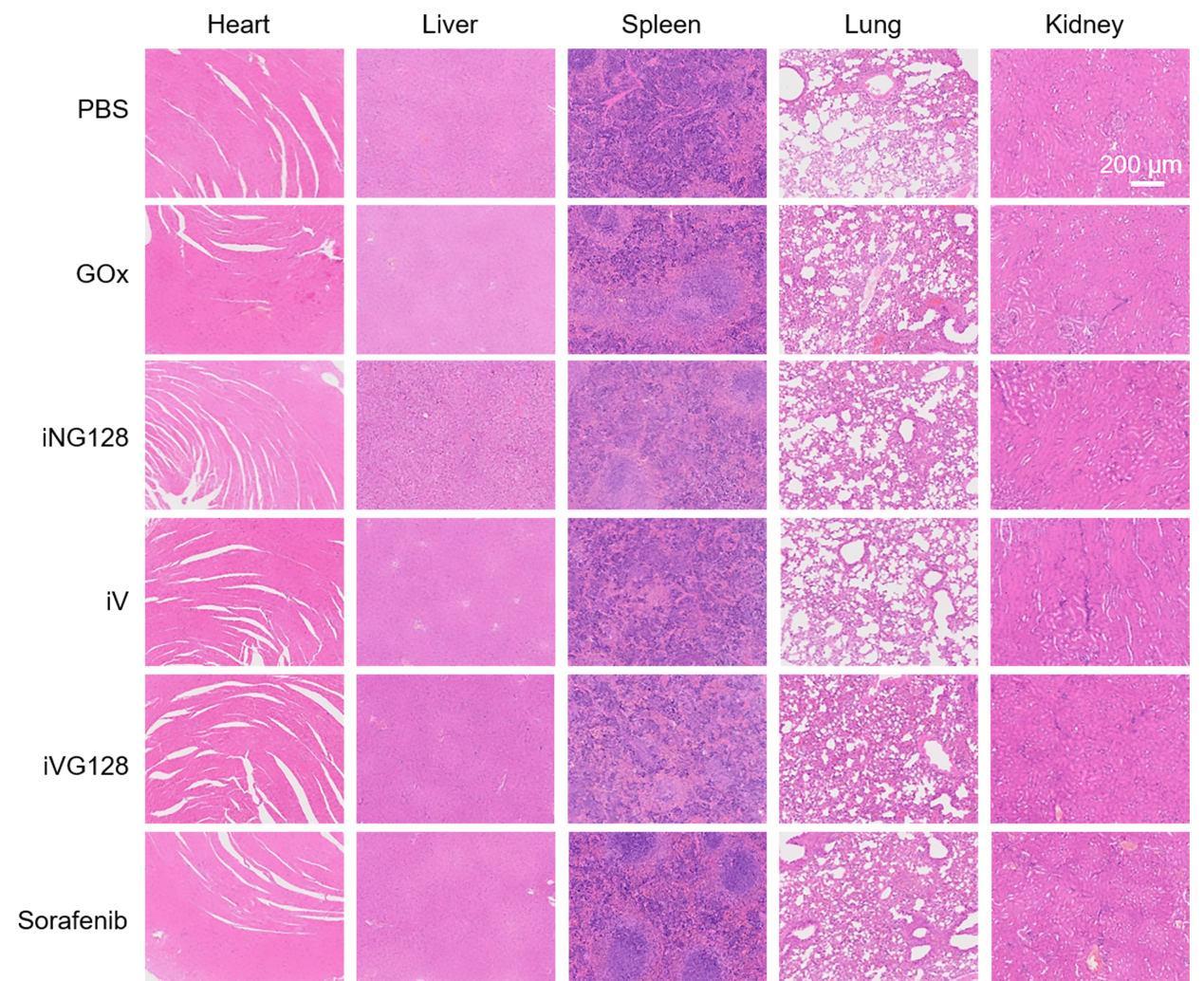


Figure S6. H&E staining of major organs in mice from different groups in the PDX model. Scale bar: 200 μm.


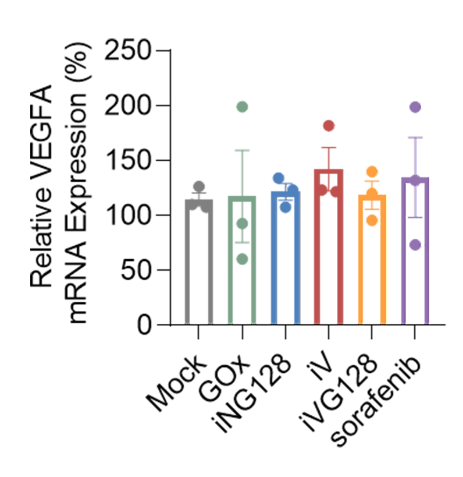


Figure S7. Hepatic VEGFA gene silencing in the PDX model.


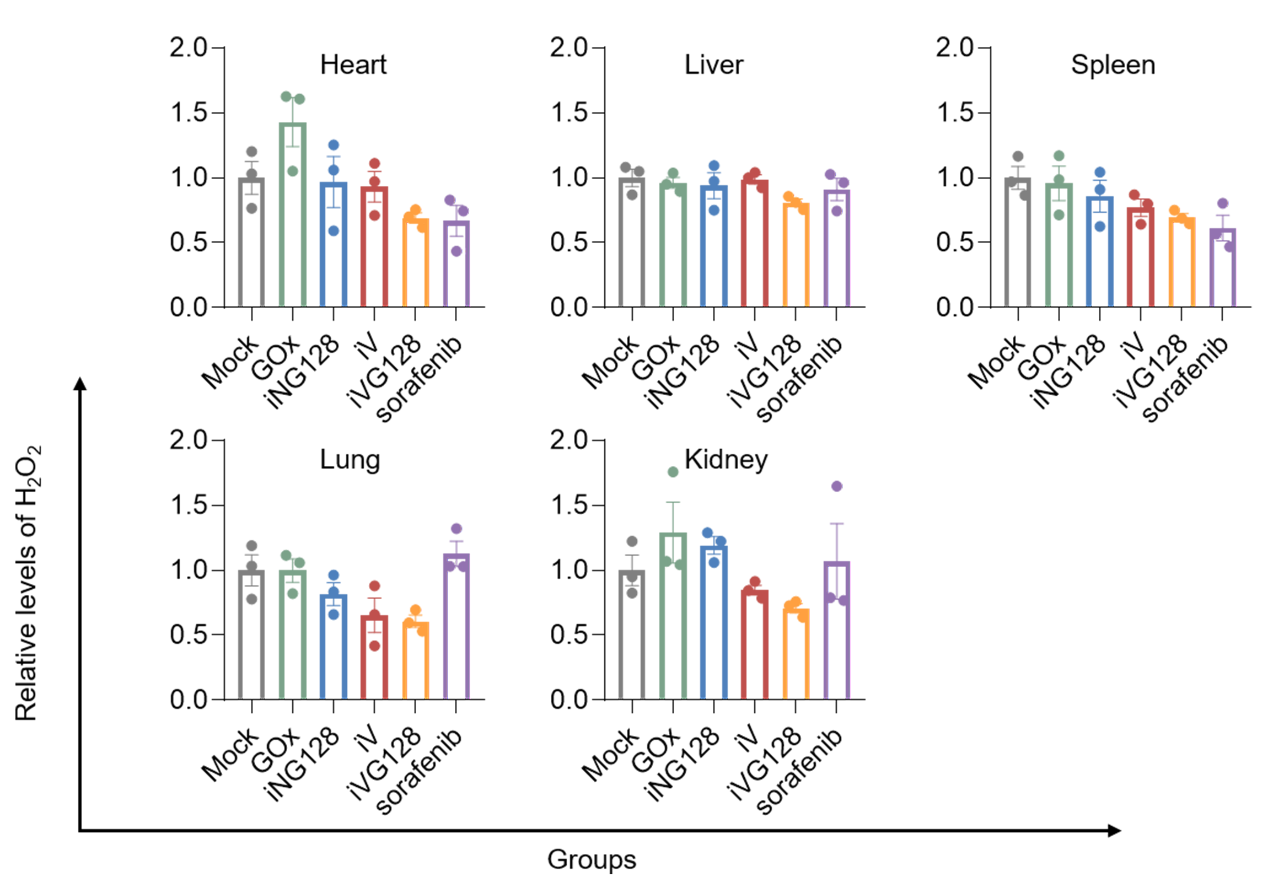


Figure S8. H₂O₂ levels in major organs after different treatments in the PDX model.


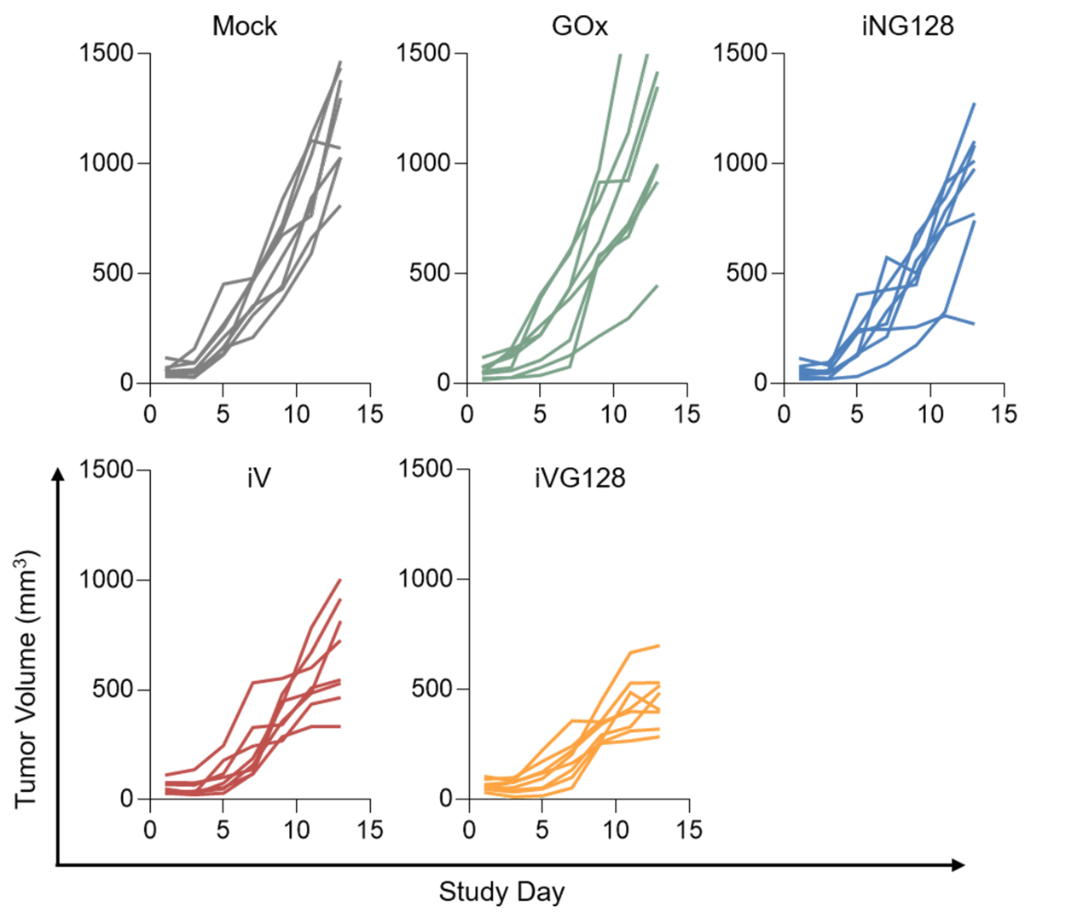


Figure S9. Tumor growth curves of mice from different groups during treatment in the CDX model.


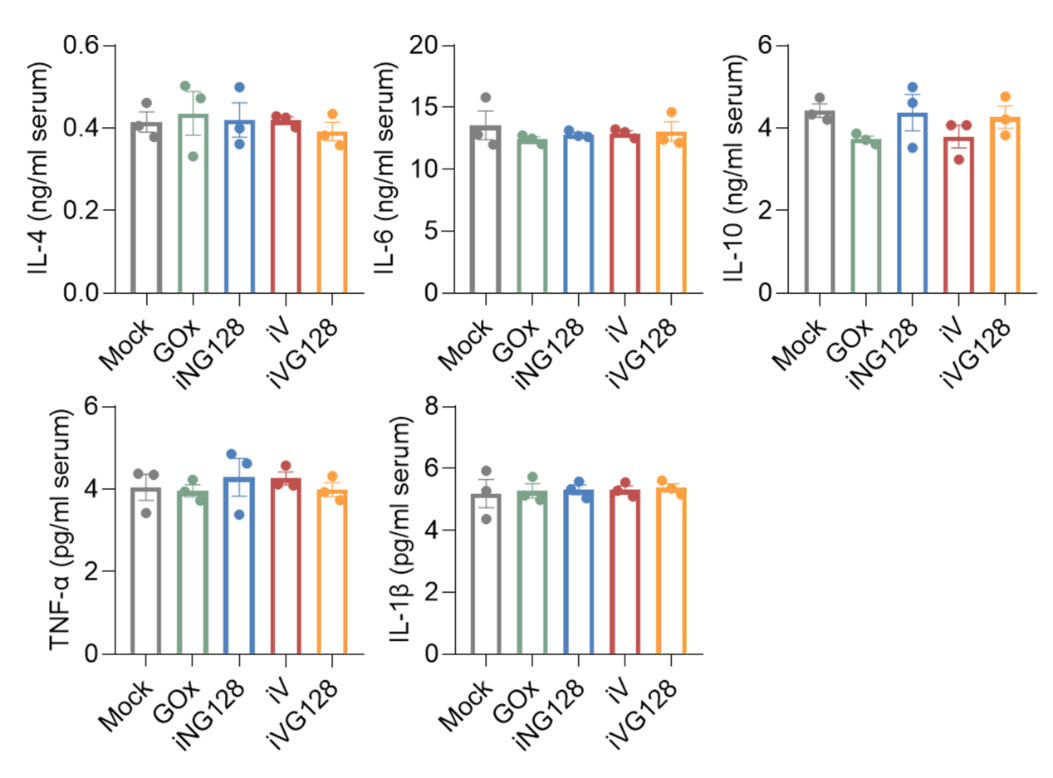


Figure S10. Systemic cytokine profiling after repeated administration. Serum levels of pro-inflammatory cytokines (IL-6, TNF-α, and IL-1β) and immunoregulatory/anti-inflammatory cytokines (IL-4 and IL-10) were quantified in mice from different treatments in the CDX model.


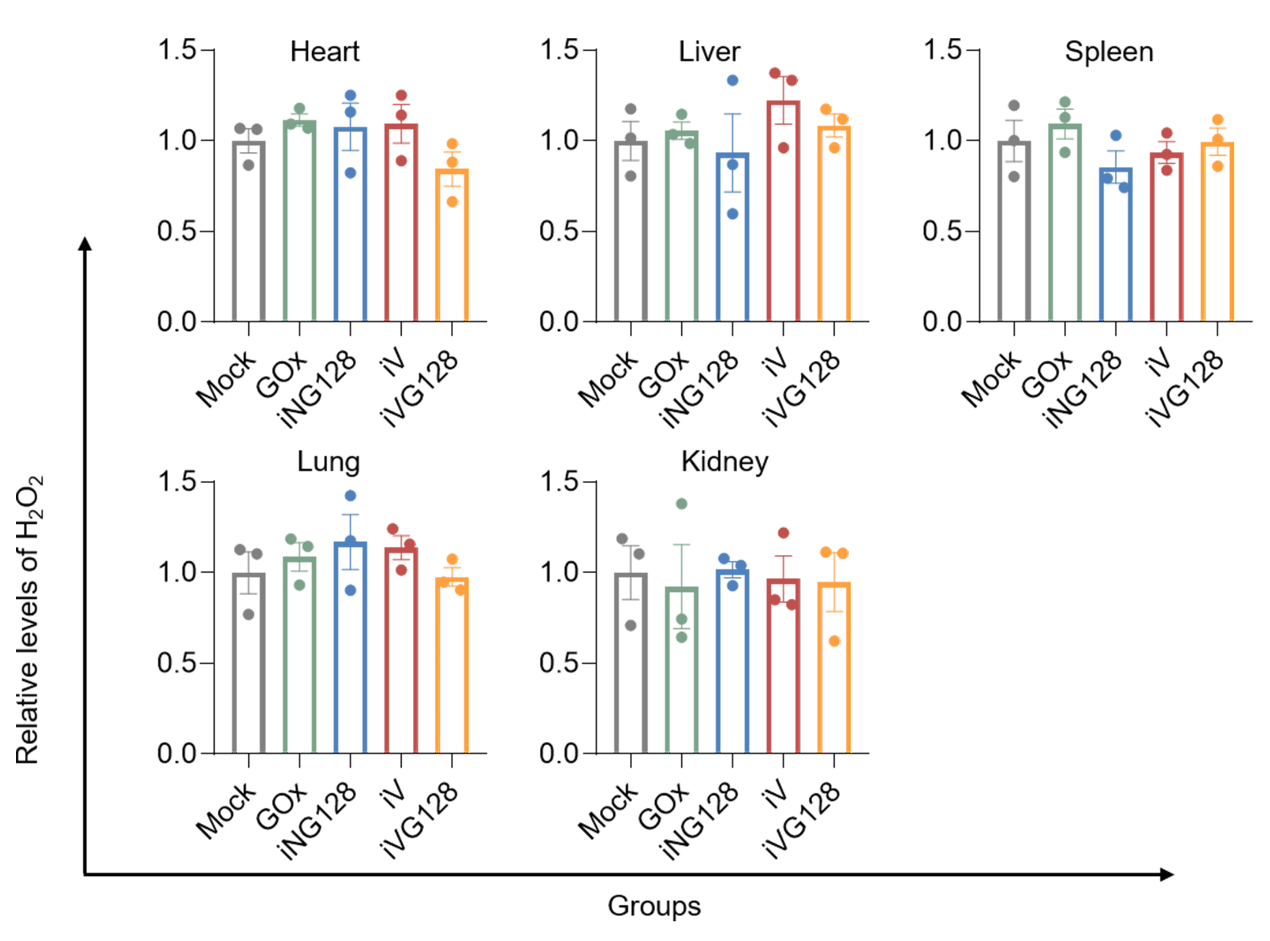


Figure S11. H₂O₂ levels in major organs after different treatments in the CDX model.


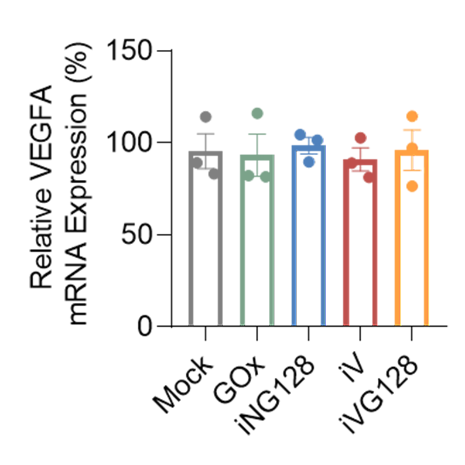


Figure S12. Hepatic VEGFA gene silencing in the CDX model.


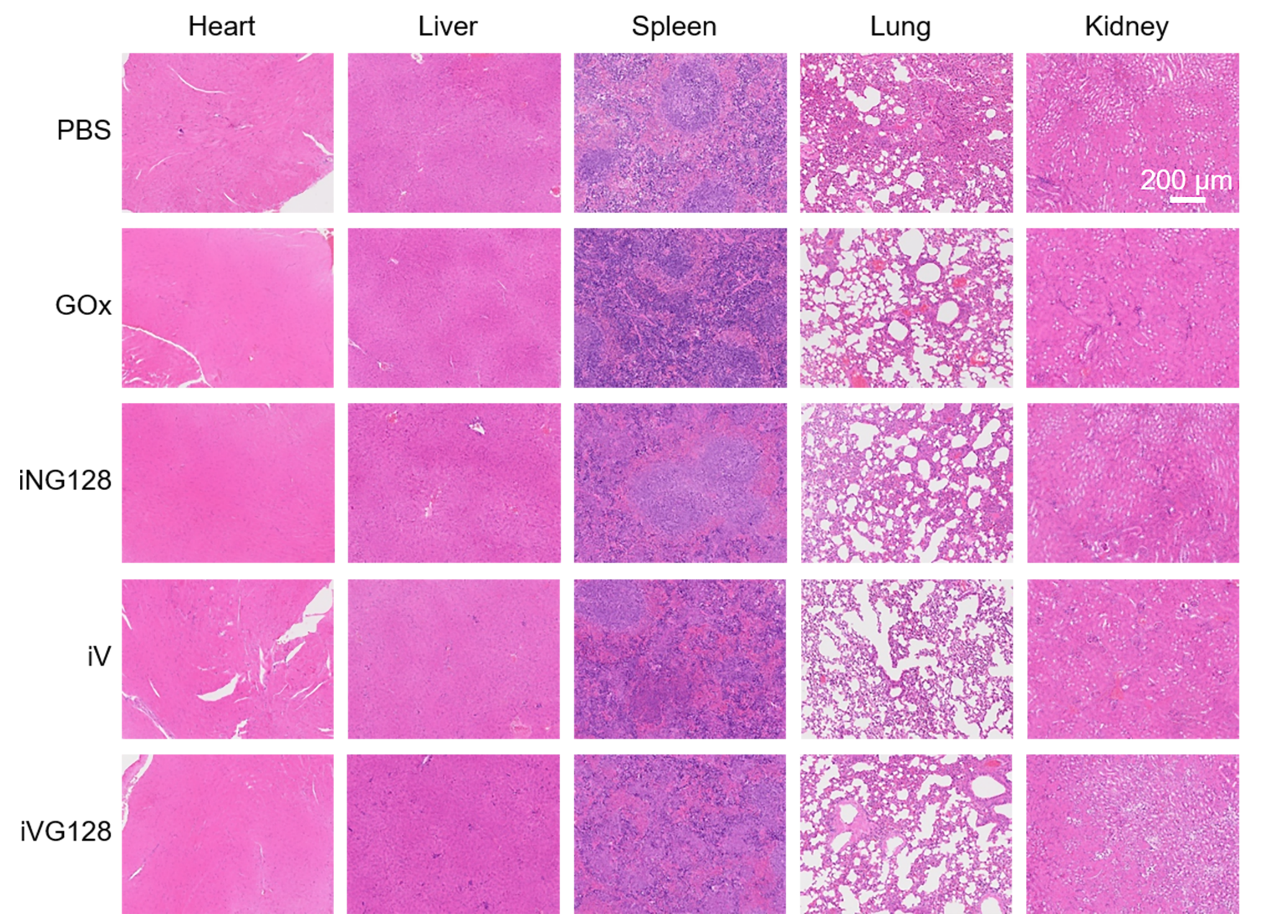


Figure S13. H&E staining of major organs in mice from different groups in the CDX model. Scale bar: 200 μm.


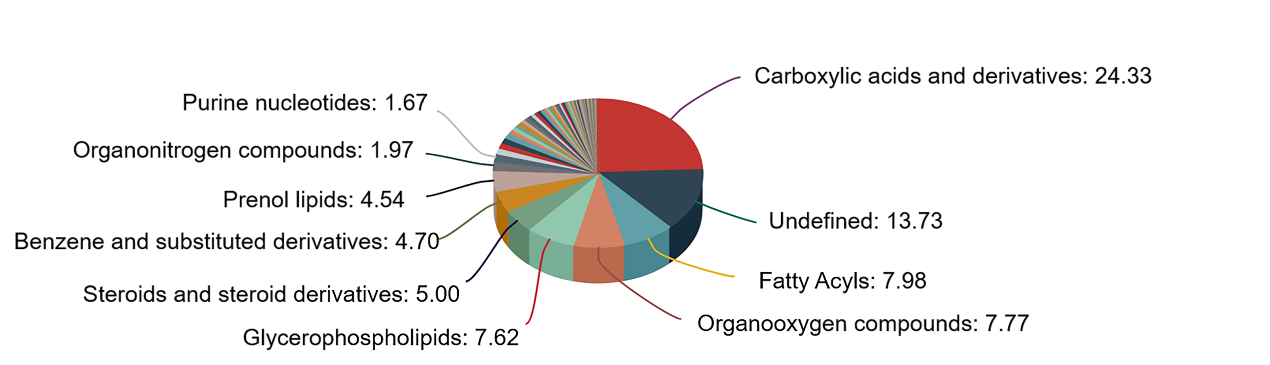


Figure S14. Proportional distribution of identified metabolites across chemical classifications. Each color block represents a distinct chemical classification category. The percentage indicates the proportion of metabolites within each category relative to the total number of identified metabolites. Metabolites without a chemical classification are defined as undefined.


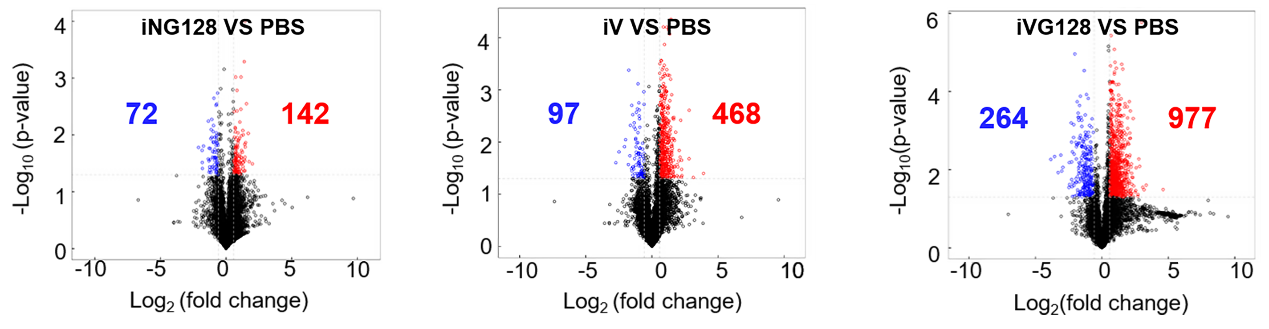


Figure S15. Volcano plots displaying differential metabolites in positive ion mode. Metabolites significantly upregulated are marked in red, while downregulated ones are shown in blue, and non-significant metabolites are shown in black.


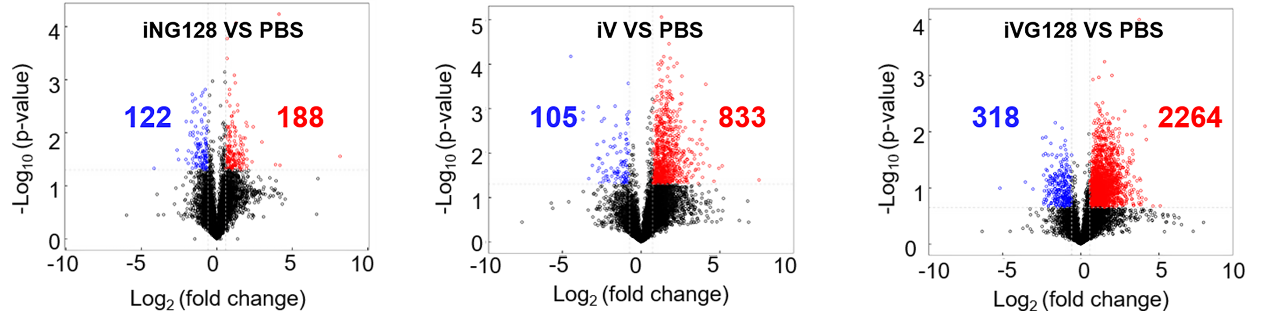


Figure S16. Volcano plot of differential metabolites in negative ion mode. Metabolites significantly upregulated are marked in red, while downregulated ones are shown in blue, and non-significant metabolites are shown in black.


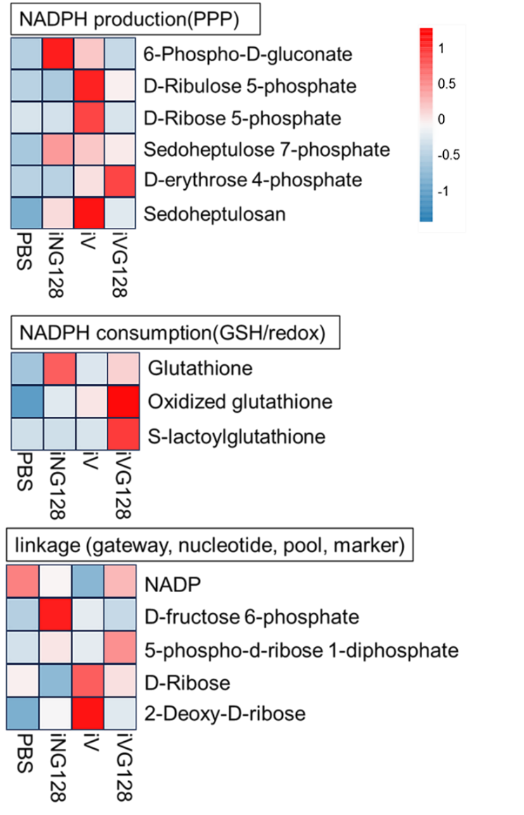


Figure S17. Metabolites are annotated by their functional role in NADPH homeostasis: NADPH production (PPP/pentose intermediates), NADPH consumption (glutathione/redox module), and other linkage nodes (glycolysis–PPP gateway, nucleotide precursor pools, and the NADP(H) cofactor pool/associated markers).


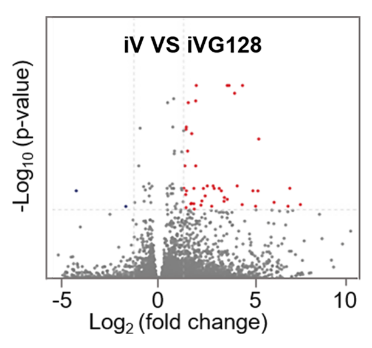


Figure S18. Volcano plot of differential gene expression between iV and iVG128. Each dot represents one gene, plotted by log2 fold change on the x-axis and −log10 adjusted p value (padj) on the y-axis. Significantly upregulated genes in iV group are shown in red, significantly downregulated genes in iV group are shown in blue, and non-significant genes are shown in gray (padj < 0.05; |log2FC| threshold as indicated by dashed lines).
